# Sex difference in evolution of cognitive decline: studies on mouse model and the Dominantly Inherited Alzheimer Network cohort

**DOI:** 10.1038/s41398-023-02411-8

**Published:** 2023-04-12

**Authors:** Reddy Peera Kommaddi, Aditi Verma, Graciela Muniz-Terrera, Vivek Tiwari, Keerthana Chithanathan, Latha Diwakar, Ruturaj Gowaikar, Smitha Karunakaran, Palash Kumar Malo, Neill R. Graff-Radford, Gregory S. Day, Christoph Laske, Jonathan Vöglein, Georg Nübling, Takeshi Ikeuchi, Kensaku Kasuga, the Dominantly Inherited Alzheimer Network (DIAN), Vijayalakshmi Ravindranath

**Affiliations:** 1 Centre for Brain Research, Indian Institute of Science, Bangalore, 560012 India; 2 Centre for Neuroscience, Indian Institute of Science, Bangalore, 560012 India; 3 Centre for Clinical Brain Sciences, The University of Edinburgh, Edinburgh, Scotland UK; 4 The Department of Social Medicine, Ohio University, Athens, OH 45701 USA; 5 Department of Neurology, Mayo Clinic Florida, Mayo Clinic College of Medicine and Science, 4500 San Pablo Road S, Jacksonville, FL 32224 USA; 6 German Center for Neurodegenerative Diseases, Munich, Germany; 7 Section for Dementia Research, Department of Cellular Neurology, Hertie Institute for Clinical Brain Research, Department of Psychiatry and Psychotherapy, University of Tübingen, Tübingen, Germany; 8 Department of Neurology, Ludwig-Maximilians-Universität München, Munich, Germany; 9 German Center for Neurodegenerative Diseases (DZNE), Munich, Germany; 10 Department of Molecular Genetics, Center for Bioresources, Brain Research Institute, Niigata University, 1-757 Asahimachi-dori, Chuo-ku, Niigata City, Niigata, 951-8585 Japan; 11 Charles F. and Joanne Knight Alzheimer Disease Research Center, Department of Neurology, Washington University School of Medicine, St. Louis, MO USA

**Keywords:** Molecular neuroscience, Hippocampus

## Abstract

Women carry a higher burden of Alzheimer’s disease (AD) compared to men, which is not accounted entirely by differences in lifespan. To identify the mechanisms underlying this effect, we investigated sex-specific differences in the progression of familial AD in humans and in *APPswe/PS1ΔE9* mice. Activity dependent protein translation and associative learning and memory deficits were examined in *APPswe/PS1ΔE9* mice and wild-type mice. As a human comparator group, progression of cognitive dysfunction was assessed in mutation carriers and non-carriers from DIAN (Dominantly Inherited Alzheimer Network) cohort. Female *APPswe/PS1ΔE9* mice did not show recall deficits after contextual fear conditioning until 8 months of age. Further, activity dependent protein translation and Akt1-mTOR signaling at the synapse were impaired in male but not in female mice until 8 months of age. Ovariectomized *APPswe/PS1ΔE9* mice displayed recall deficits at 4 months of age and these were sustained until 8 months of age. Moreover, activity dependent protein translation was also impaired in 4 months old ovariectomized *APPswe/PS1ΔE9* mice compared with sham female *APPswe/PS1ΔE9* mice. Progression of memory impairment differed between men and women in the DIAN cohort as analyzed using linear mixed effects model, wherein men showed steeper cognitive decline irrespective of the age of entry in the study, while women showed significantly greater performance and slower decline in immediate recall (LOGIMEM) and delayed recall (MEMUNITS) than men. However, when the performance of men and women in several cognitive tasks (such as Wechsler’s logical memory) are compared with the estimated year from expected symptom onset (EYO) we found no significant differences between men and women. We conclude that in familial AD patients and mouse models, females are protected, and the onset of disease is delayed as long as estrogen levels are intact.

## Introduction

Sex related differences have been observed in the progression of Alzheimer’s disease (AD) [1] and the prevalence of AD is greater in women than men in European and American patients [2, 3]. Globally, the number of women living with dementia including AD is more than that of men [4], which has been attributed to longer lifespan (4–5 years) in women [5]. Women (65 years or older) are at a greater risk of developing late-onset AD (LOAD) [6–8]. Cross-sectional and longitudinal studies have revealed faster cognitive decline [9] and widespread atrophy in brain areas in early-onset AD (EOAD) [10–17]. Women EOAD subjects displayed greater cognitive impairment and atrophy than men [18]. Preclinical individuals carrying autosomal dominant AD (ADAD) mutations showed memory deficits [19–21]. Neurological examination findings in AD (AD-NEF) from mutation carriers and non-carriers from the Dominantly Inherited Alzheimer Network (DIAN) revealed that AD-NEF are associated with a rapid cognitive decline and higher hippocampal atrophy [22]. Sex differences in the genetic make-up of resilience and multiple sex-specific molecular mechanisms may underlie resilience to AD pathology [23]. Perimenopausal and postmenopausal women aged 40 to 60 had more AD endophenotype than premenopausal women [24]. Further, women are well protected from stroke [25] and other neurodegenerative diseases relative to men until menopause, however, when the female sex hormone levels decline sharply at menopause, the clinical outcome is worse.

At the epidemiological level, it is crucial to comprehend the sex-specific differences in AD in relation to several elements, including longevity, survival bias, and comorbidities [26]. The risk and development of AD can differ between men and women depending on sociocultural and biological factors [26]. Menopause, oophorectomy, and androgen-deprivation therapy are sex-specific AD risk factors that cause cognitive decline [26]. Further, sex hormones play a critical role in sex specific differences in the brain [27]. However, the molecular mechanisms underlying the role of endogenous estradiol, social behavioral deficits, and the higher burden of AD pathogenesis in women remain unknown. Estrogen is neuroprotective [28] and has a protective effect on the vasculature [29]. Estrogens facilitate hippocampal synaptic plasticity and memory formation [30–33]. Although several animal models have been developed for simulating the state of estrogen depletion and testing the role of estrogen in synaptic plasticity, the ovariectomized (OVX) animals are a well-established model [34–36]. Depletion of estrogen levels in the brain may confer greater susceptibility to age-related neurodegenerative diseases [37]. Further, estrogen levels are significantly decreased in postmortem frontal cortex brain lysates of AD subjects compared with non-AD subjects [38]. In fact, decrease in estrogen levels seen during the transition from perimenopause to menopause coincides with increased β-amyloid (Aβ) deposition [38, 39]. While several studies have attempted to explain the effects of sex differences in AD, the underlying molecular pathways have not been fully explored. Moreover, the potential relationship between the female sex hormone, estrogen and memory impairments associated with AD is yet to be understood.

Akt signaling cascade plays a critical role in neurotransmission and synaptic plasticity [40–42]. The mTOR signaling pathway is the major nutrient-sensitive regulator for cell growth and metabolism [43]. The mTOR signaling cascade is implicated in the regulation of activity dependent mRNA translation at the synapses, which is induced by synaptic activity and required for effective LTP and memory formation [44]. Activation of Akt-mTOR signaling cascade is critical for new protein synthesis at the synapse. Dysregulation of Akt/mTOR has been reported using postmortem brain tissue from AD subjects and in AD animal models [45]. But, how Akt1 and mTOR signaling pathways regulate activity dependent new protein synthesis at synapse has not yet been explored in female AD mouse models.

To investigate this phenomenon, we used a mouse model of AD (*APPswe/PS1ΔE9*) and examined the molecular mechanisms underlying the pathogenesis and progression of AD. To understand how the findings in the mouse model extrapolate to humans, we analyzed the clinical longitudinal data from the DIAN study, in which participants carry one of the familial mutations in APP, PSEN1 or PSEN2 genes. The rationale for examining findings made in participants carrying familial AD mutations was that these participants show cognitive dysfunctions much earlier and therefore we would have window to look at differences between men and women carrying mutations in their third to fourth decade of life. Non-carriers are siblings of mutation carriers with no mutations considered as a control group. Importantly, this provides us an insight into the progression of the disease in premenopausal women, who have estrogen levels that are relatively intact.

## Materials and methods/subjects

### Reagents

Analytical grade chemicals and reagents were procured from Sigma-Aldrich Chemical Company (St Louis, MO, USA). L-[^35^S]-Methionine was purchased from Perkin Elmer Inc, USA. Amytracker 520 was purchased from Ebba Biotech, Sweden. Mouse monoclonal anti-β-tubulin (Cat. No. T4026, RRID: AB_477577) was purchased from Sigma-Aldrich Chemical Company (St Louis, MO, USA). Anti-4E-BP1, phospho (Thr37/Thr46) (Cat. No. 2855, RRID: AB_560835), Rabbit Anti-Akt1 (Cat. No. 2938, RRID: AB_915788), Phospho-Akt1 (Ser473) (Cat. No. 4060, RRID: AB_2315049), Anti-4E-BP1 (53H11) (Cat. No. 9644, RRID: AB_2097841), Phospho-Akt (Thr308) (D25E6) (Cat. No.13038, RRID: AB_262944), Anti-mTOR (L27D4) (Cat. No. 4517, RRID: AB_1904056), Anti-GSK-3β (3D10) (Cat. No. 9832, RRID: AB_10839406), Phospho-GSK-3β (Ser9) (Cat. No. 9336, RRID: AB_331405), Phospho-p70 S6 Kinase (Thr389) (1A5) (Cat. No. 9206, RRID: AB_2285392), p70S6 Kinase (Cat. No. 9202, RRID: AB_331676), Phospho-mTOR (Ser2448) (D9C2) (Cat. No. 5536, RRID: AB_10691552) were purchased from Cell Signaling Technology, Inc, USA. Horseradish peroxidase-conjugated secondary antibodies were purchased from Vector Laboratories, Inc. (Burlingame, CA, USA).

### Animals

The *APP*_*Swe*_*/PS1ΔE9* (*APP/PS1*) double transgenic mice on C57BL/6 J background were procured from the Jackson Laboratory, USA (https://www.jax.org/strain/005864; RRID: MMRRC_Stock_No: 034832-JAX). All experiments were conducted with *APP/PS1* and littermate wild-type mice. Wild type (WT) and *APP/PS1* double transgenic mice were bred at the Institutional Central Animal Facility and all mice were cared by the central animal facility members including veterinary physician and staff. All mice were housed in a temperature-controlled room on a 12 h light/12 h dark cycle and these rooms were maintained under sterile and pathogen-free conditions. All mice had *ad libitum* access to food and water. All experimental protocols and procedures were approved by the Institutional Animal Care and Use committee and are in accordance with the Guide for the care and Use of Laboratory Animals. All efforts were made to reduce suffering of mice and the number of mice used for our required experiments.

Observed power of analysis is known to be inversely proportional to observed *p*-value. Variability in transgenic *APP/PS1* expression across mice results in variation in the observed biochemical parameters. To account for this impact and rule out the possibility of litter-specific effects, 4–8 mice per group were selected from separate litters and processed individually for each biochemical assay. For behavioral studies, the number of animals to be utilized was based on behavioral tests conducted in several labs, and 6–10 mice per genotype were selected as the sample size.

Data inclusion and exclusion. No samples were excluded from the experiments or analysis.

Randomization and blinding. All the animal experiments were designed and followed in compliance with the ARRIVE guidelines and applying double-blinded analysis when possible. In experiments involving wild type and *APP/PS1* mice, the animals were assigned randomly to the respective groups based on the genotype.

In some experiments, female mice (2–2.5 months old) were anesthetized by intraperitoneal administration of 100 mg ketamine and 25 mg xylazine/kg body weight. The animal was fixed on surgical board with heating pad to maintain body temperature and an incision of about 2–3 cm was made in the lower abdomen for ovariectomy. Ovaries were removed carefully, and the abdomen was sutured. In the sham-operated mice, a similar incision was made, and the abdomen was sutured without removing ovaries. Ovariectomized and sham-operated mice were used for experimentation after 2 and 6 months. During sacrifice, atrophy of uterus and lack of ovaries were examined to ensure success of ovariectomy.

### Contextual fear conditioning

Contextual fear conditioning (cFC) behavior was assessed as reported previously [46] and detailed protocol presented as supplementary methods.

### Preparation of synaptosomes

Synaptosomal fractions were isolated as described previously [45] and detailed protocol provided as supplementary methods.

### Immunoblotting

Equal amounts of synaptosomes were resolved on sodium dodecyl sulfate polyacrylamide gel electrophoresis and transferred to a polyvinylidene difluoride membrane by electroblotting. Immunoblots were blocked in 5% bovine serum albumin for 1 hour at room temperature and immunoblotted with respective primary antibodies and incubated at 4 °C overnight. The following day, all immunoblots were washed and incubated at room temperature in respective secondary antibodies. Immunoreactive bands were detected using enhanced chemiluminescence (Clarity Western ECL blotting substrate, Bio-Rad). Immunoreactive signals were acquired using the Bio-Rad Chemidoc-XRS and analyzed with Imagelab software (Bio-Rad).

### Isolation of synaptoneurosomes and L-[^35^S]-methionine incorporation assay

Isolation of synaptoneurosomes from mouse hippocampal tissue and L-[^35^S]-methionine incorporation assay were performed as described previously [45] and detailed protocol provided as supplementary methods.

### Statistical analyses from mice studies

Statistical analyses were performed using GraphPad Prism (Prism 7.01, GraphPad Software Inc, La Jolla, CA, USA). Continuous variables were checked for normality using Shapiro-Wilk test and then an independent t-test or Mann–Whitney *U*-test was used appropriately to compare between two groups. Statistical comparisons more than two groups were performed using ANOVA with Tukey’s post hoc test. Results are represented as mean ± standard error of the mean. A *p*-value of <0.05 was considered statistically significant. No statistical methods were used to predetermine sample sizes.

### DIAN data analyses

Both longitudinal and cross-sectional data on psychometric tests were obtained from the DIAN study (DIAN Data Freeze 12). Research involving human participants was done in accordance with the guidelines provided by the Institutional Human Ethics Committee (IHEC; approval# 10/1/2015). The age ranges of mutation carriers are men (19 years to 67 years, SD ± 10.79 years), women (18 years to 67 years, SD ± 10.84 years), and non-carriers are men (18 years to 66 years, SD ± 10.1 years), women (18 years to 69 years, SD ± 11.4 years). The characteristics of DIAN participants are summarized in Supplementary Table 1. To assess cognitive decline, analysis was performed on longitudinal Mini-Mental State Exam (MMSE) data. Analysis was also performed on longitudinal data from tests for episodic memory including immediate and delayed Wechsler’s logical memory test and immediate and delayed word list recall. Linear mixed effects models (lme) were fitted between scores of various cognitive examinations and age for men and women subjects in mutation carriers and non-carrier groups using the statistical computing language, R (lme ‘R’ package). Models were structured as a function of age. The intercept was placed at the average age at study entry. However, as participants enter the study at different ages, to account for between person differences in baseline age, we still controlled the intercept and slope for age at study entry. Intercept and rate of change were adjusted for age at baseline and sex. The linear fit was used to determine the correlation and the rate of changes in cognitive score with aging. We fitted a series of linear mixed effects models to cognitive scores from each of the tests available in the DIAN study.

The equations are as follows:

$$Y_{it} = \alpha _{i0} + \alpha _{i1}EYO_{it} + \varepsilon _{it};$$

$$\alpha _{i0} = \alpha _0 + \beta _0sex_i + \gamma _0Age_{i0} + u_{0i};$$

$$\alpha _{i1} = \alpha _1 + \beta _1sex_i + \gamma _1Age_{i0} + u_{1i};$$

$${{{\mathrm{Where}}}}\;{{{\mathrm{the}}}}\;{{{\mathrm{error}}}}\;{{{\mathrm{terms}}}}\left[ {\begin{array}{*{20}{c}} {u_{0i}} \\ {u_{1i}} \end{array}} \right]\sim N\left( {0,\;T} \right),\;with\;T = \left[ {\begin{array}{*{20}{c}} {\tau _{00}} & {\tau _{01}} \\ {\tau _{01}} & {\tau _{11}} \end{array}} \right]\;{{{\mathrm{and}}}}\;\varepsilon _{it}\sim N\left( {0,\;\sigma ^2} \right).$$

Here, *Y*_*it*_ represents the result of the cognitive test Y for individual i at time t; and *α*_*i*0_ and *α*_*i*1_ individual i’s intercept and linear rate of change per year closer to the EYO. These are modeled as a function of population mean values *α*_0_ and *α*_1_ respectively, and the individual’s age at study entry and sex. The error term *ε*_*it*_ represents within-individual random error whereas *u*_0*i*_ and *u*_1*i*_ are the between-individual random effects that estimate the difference, after controlling for age at study entry and sex, between the population mean intercept and rate of change (*α*_0_ and *α*_1_) and the individual intercept and rate of change respectively.

Cognitive scores were aligned as a linear function of the estimated year from expected symptom onset (EYO) at each visit, setting the intercept at EYO = 0. The estimated years of symptom onset were calculated as the age of the participant at the time of the study assessment minus the age of the parent at symptom onset [47]. Both the intercept and rate of change were adjusted for baseline age and sex. Independent analyses were conducted for the subsamples of mutation carrier and non-carrier participants, splitting each of the subsamples by CDR status (CDR = 0 and CDR > 0). All models were estimated using the lme function of the nlme package, which uses restricted maximum likelihood estimation and assume missing data are missing at random.

## Results

### Age dependent impairment of contextual fear memory in *APP/PS1* mice

**Fig. 1:**
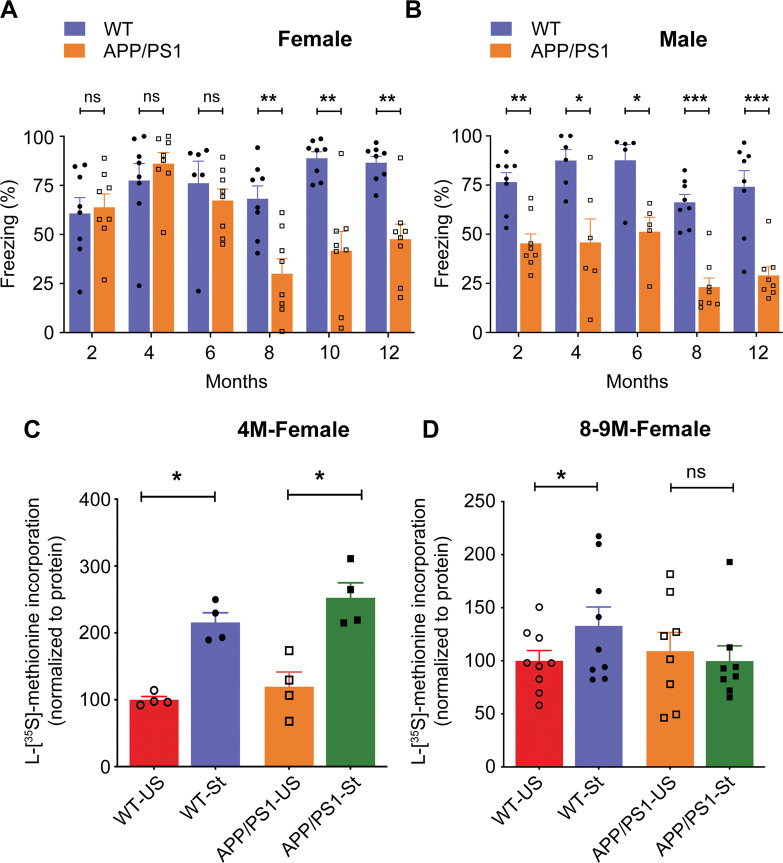
Impairment in recall memory after contextual fear conditioning depends on sex and age of *APP/PS1* mice. **A** Histograms are showing the freezing response of female mice at different ages. Freezing response was assessed by contextual fear conditioning as percentage of immobility after 24 h of training and no significant difference was observed in 2 M (*t* = *0.2923; df* = 14; *p* = 0.7744; Hedges’ g (95% CI) 0.14 (−0.79, 1.06)), 4 M (*p* = 0.4255; Hedges’ g (95% CI) 0.39 (−0.54, 1.32)) and 6 M (*p* = 0.1419; Hedges’ g (95% CI) −0.37 (−1.37, 0.63)) old *APP/PS1* female mice compared with WT female mice. Impairment in freezing response was observed in 8 M (*t* = *3.794; df* = 14; *p* = 0.0020; Hedges’ g (95% CI) −1.79 (−2.9, −0.63)), 10 M (*t* = 4.538*; df* = 14; *p* = 0.0005; Hedges’ g (95% CI) −2.14 (−3.33, −0.90)), and 12 M (*t* = *4.65; df* = 14; *p* = 0.0004; Hedges’ g (95% CI) -2.19 (−3.40, −0.94)) old *APP/PS1* female mice compared with age matched WT female mice. Statistical analysis: Two-sided *t*-test or Mann-Whitney U-test was applied to compare WT versus *APP/PS1* mouse groups. In all panels, reported values are mean ± s.e.m. *P < 0.05. **A**, **B** *n* = 5–8 mice per group. **B** Histograms are showing the freezing response of male mice at different ages, and *APP/PS1* male mice exhibit significantly decreased freezing response than the WT male from 2 months onwards. WT versus *APP/PS1* male mice, 2 M (*t* = *4.594; df* = 14; *p* = 0.0004; Hedges’ g (95% CI) −2.17 (−3.37, −0.92)), 4 M (*t* = *3.168; df* = 10; *p* = 0.01; Hedges’ g (95% CI) −1.68 (−2.93, −0.38)), 6 M (*p* = 0.0317; Hedges’ g (95% CI) −1.90 (−3.32, −0.42)), 8 M (*p* = 0.0002; Hedges’ g (95% CI) −3.33 (−4.85, −1.78)), and 12 M (*t* = *4.824; df* = 14; *p* = 0.0003; Hedges’ g (95% CI) −2.28 (−3.50, −1.01)). Statistical analysis: Two-sided *t*-test or Mann–Whitney U-test was used to compare WT versus *APP/PS1* mouse groups. In all panels, reported values are mean ± s.e.m. *P < 0.05. **A**, **B** *n* = 5–8 mice per group. Local activity dependent protein translation at the synapse is significantly affected in *APP/PS1* female mice at 8 months but not at 4 months of age. Synaptoneurosomes from WT and *APP/PS1* female mice were stimulated with or without KCl in the presence of 50 μCi L-[^35^S]-methionine at 37 °C for 15 min and newly synthesized proteins (protein translation) were measured by L-[S^35^]-methionine incorporation assay. **C** KCl stimulated local protein translation in synaptoneurosomes was not affected between WT and *APP/PS1* female mice at 4 months of age (WT-US versus WT-St, *t* = 6.894*; df* = 3; *p* = 0.003; Hedges’ g (95% CI) 4.61 (1.78, 7.40); *APP/PS1*-US versus *APP/PS1*-St, *t* = *3.042; df* = 3; *p* = 0.028; Hedges’ g (95% CI) 2.59 (0.68, 4.42)). Statistical comparisons from each group (unstimulated versus stimulated) were calculated using paired, one-sided, student *t-*test. *n* = 4 mice per group. **D** Stimulation of local protein translation in synaptoneurosomes in the presence of KCl was impaired in *APP/PS1* female mice with comparison to WT female mice at 8 months of age (WT-US versus WT-St (*t* = *2.960; df* = 8; *p* = 0.009; Hedges’ g (95% CI) 0.72 (−0.19, 1.63))*; APP/PS1*-US *versus APP/PS1*-St (*t* = *0.341; df* = 7; *p* = 0.372; Hedges’ g (95% CI) −0.19 (−1.11, 0.74))). Statistical comparisons from each group (unstimulated versus stimulated) were calculated using paired, one-sided, student *t-*test. All values normalized to unstimulated WT group. Data is represented as mean ± s.e.m. (*n* = 8–9 mice per group) and *denotes values significantly different from corresponding controls (*p* < 0.05).

In order to examine potential sex-related differences in learning and memory as a function of age and the presence of amyloidosis, we assessed the recall deficits, if any, on cFC in male and female *APP/PS1* mice at different ages (2, 4, 6, 8, 10, and 12 months). At 2 months of age (*t* = *0.2923; df* = 14; *p* = 0.7744), 4 months of age (*p* = 0.4255), and 6 months of age (*p* = 0.1419), *APP/PS1* female mice behaved like WT and did not display any recall deficits (Fig. 1A). However, *APP/PS1* male mice showed these recall deficits as early as 2 months of age (*t* = *4.594; df* = 14; *p* = 0.0004) (Fig. 1B) and these recall deficits persisted at 4 months of age (*t* = *3.168; df* = 10; *p* = 0.01), 6 months of age (*p* = 0.0317), 8 months of age (*p* = 0.0002), and 12 months of age (*t* = *4.824; df* = 14; *p* = 0.0003) (Fig. 1B). In the present study, we did not conduct recall memory task using male mice at 10 months of age. Remarkably, we found that at 8 months of age, *APP/PS1* female mice showed significant recall deficits as compared to WT (*t* = *3.794; df* = 14; *p* = 0.002) (Fig. 1A; 10 months of age (*t* = *4.538; df* = 14; *p* = 0.0005)), which was sustained until 12 months of age (*t* = *4.650; df* = 14; *p* = 0.0004) (Fig. 1A). Our results provide evidence that deficits in recall after cFC were not observed in young *APP/PS1* female mice but emerged significantly at 8 months of age and later, while in the corresponding male mice the recall deficits were seen from 2 to 12 months of age.

### Impairment of synaptic activity dependent protein translation is delayed in hippocampus of *APP/PS1* female mice

The activity dependent protein translation at the synapse is essential for learning and memory and synaptic plasticity. Here we sought to test whether activity dependent protein translation is affected in *APP/PS1* female mice of different age groups by measuring [^35^S]-L-methionine incorporation in newly synthesized proteins. Remarkably, we found that incorporation of [^35^S]-L-methionine by KCl stimulation, which is indicative of protein translation, was unaffected in *APP/PS1* female mice at 4 months of age (*WT-US* versus *WT-St* (*t* = 6.894*; df* = 3; *p* = 0.003)*; APP/PS1-US versus APP/PS1-St* (*t* = 3.042*; df* = 3; *p* = 0.028)) (Fig. 1C), while this was absent in young *APP/PS1* male mice [45] indicating that activity dependent protein translation is intact in *APP/PS1* female mice at early age. However, at 8–9 months of age, KCl stimulated [^35^S]-L-methionine incorporation was affected similarly in both *APP/PS1* male [45] and female mice (*WT-US* versus *WT-St* (*t* = 2.960*; df* = 8; *p* = 0.009*); APP/PS1-US versus APP/PS1-St* (*t* = 0.341*; df* = 7; *p* = 0.372)) (Fig. 1D) indicating that no activity dependent translation occurred in the synaptoneurosomes of *APP/PS1* mice.

### Effect of ovariectomy on recall of contextual fear conditioning in APP/PS1 female mice

**Fig. 2:**
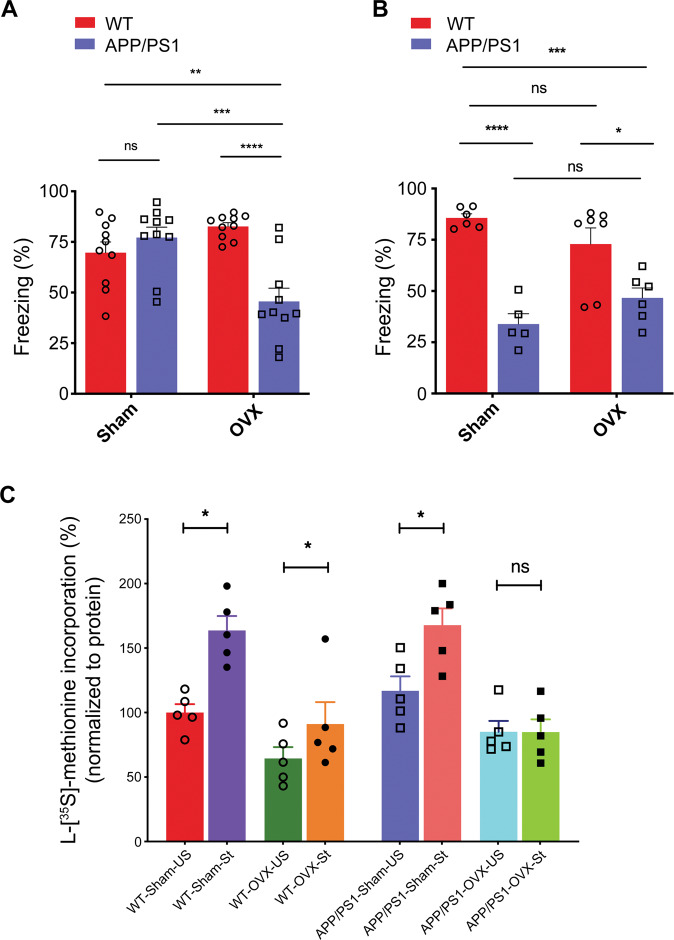
Ovariectomy persuaded recall deficits and induced impairment of local activity dependent protein translation after contextual fear conditioning in *APP/PS1* female mice at 4 months of age. Sham or ovariectomized WT and *APP/PS1* female mice were subjected contextual fear conditioning at 4- and 8-months age and assessed their recall memory as described in methods section. **A** Impairment in freezing response was observed in OVX *APP/PS1* female mice following contextual fear conditioning at 4 months of age with comparison to all other groups as examined. (WT-Sham versus APP/PS1-Sham, *p* = 0.7251; WT-Sham versus WT-OVX, *p* = 0.2798; APP/PS1-Sham versus APP/PS1-OVX, *p* = 0.0005; WT-Sham versus APP/PS1-OVX, *p* = 0.0086; WT-OVX versus APP/PS1-OVX, *p* < 0.0001). Partial Eta squared = 0.353. *n* = 10 mice per group. **B** Sham or OVX *APP/PS1* female mice exhibit significantly decreased freezing response than the sham or OVX WT female mice at 8 months of age. *n* = 5–7 mice per group. Statistical comparison is performed using two-way ANOVA. (WT-Sham versus APP/PS1-Sham, *p* < 0.0001; WT-Sham versus WT-OVX, *p* = 0.3835; APP/PS1-Sham versus APP/PS1-OVX, *p* = 0.4522; WT-Sham versus APP/PS1-OVX, *p* = 0.0005; WT-OVX versus APP/PS1-OVX, *p* = 0.0146). Partial Eta squared=0.197. Data are presented as mean ± s.e.m. Statistical significance indicated as *p < 0.05, **p < 0.01, ***p < 0.001, ****p < 0.0001. *n* = 5–7 mice per group. Protein translation in synaptoneurosomes from sham or OVX WT and *APP/PS1* female mice after KCl stimulation was measured as L-[^35^S]-methionine incorporation. **C** L-[^35^S]-methionine incorporation by KCl stimulation was significantly increased in synaptoneurosomes from sham WT or *APP/PS1* female mice (4 months) than unstimulated synaptoneurosomes. KCl stimulated protein translation was significantly impaired in synaptoneurosomes from OVX *APP/PS1* female mice (4 months) than unstimulated OVX *APP/PS1* female mice (4 months). Statistical comparisons from each group (unstimulated versus stimulated) were calculated using paired, one-sided, student *t-*test. All values normalized to unstimulated WT-sham group. (WT-Sham-US versus WT-Sham-St, *t* = 5.442*; df* = 4; *p* = 0.003; Hedges’ g (95% CI) 2.79 (1.01, 4.51); WT-OVX-US versus WT-OVX-St, *t* = *2.649; df* = 4; *p* = 0.029; Hedges’ g (95% CI) 0.79 (−0.41, 1.95); APP/PS1-Sham-US versus APP/PS1-Sham-St, *t* = *6.62; df* = 4; *p* = 0.001; Hedges’ g (95% CI) 1.69 (0.27, 3.04); APP/PS1-OVX-US versus APP/PS1-OVX-St, *t* = *0.06526; df* = 4; *p* = 0.476; Hedges’ g (95% CI) −0.01 (−1.13, 1.10)). Data are presented as mean ± s.e.m. Statistical significance indicated as *p < 0.05. *n* = 5 mice per group.

To test the effect of ovariectomy on hippocampal dependent learning and memory, WT and *APP/PS1* female mice were ovariectomized (OVX) at 2 months of age and then housed for a further period of 2 or 6 months. Next, we evaluated the recall after cFC in WT and *APP/PS1* from sham and OVX female mice. At 4 months of age, OVX *APP/PS1* female mice showed significant recall deficits as compared to sham *APP/PS1* female mice (Interaction: F (1, 36) *=* 19.64*, p* < 0.0001; OVX: F (1, 36) = 3.438*, p* = 0.0719; Genotype: F (1, 36) = 8.732*, p* = 0.0055) (Fig. 2A), and this observation was similar to that in *APP/PS1* male mice [46]. However, recall deficits after cFC were not observed in sham *APP/PS1* female mice compared with those in sham WT female mice at this age (Fig. 2A). Further, OVX *APP/PS1* female mice exhibited significant recall deficits in comparison to sham or OVX WT female mice (Fig. 2A). Additionally, sham and OVX *APP/PS1* female mice displayed significant recall deficits in comparison to sham and OVX WT female mice at 8 months of age (Interaction: F (1, 20) = 4.904*, p* = 0.0386; OVX: F (1, 20) *=* 1.991e-005*, p* = 0.9965; Genotype: F (1, 20) = 46.05*, p* < 0.0001) (Fig. 2B). Our results indicate that ovariectomy enhances memory impairments in *APP/PS1* female mice at early age.

### Effect of ovariectomy on synaptic activity dependent protein translation in hippocampus of *APP/PS1* female mice

Estrogen can contribute to the regulation of local protein translation and it can rapidly stimulate the phosphorylation of Akt and 4E-BP1 [48]. Here, we sought to determine whether ovariectomy can regulate activity dependent protein translation in *APP/PS1* female mice. To test this, we examined the effect of ovariectomy on new protein synthesis as measured by L-[^35^S]-methionine labeling. We found that stimulation of synaptoneurosomes with KCl from sham or OVX WT female mice displayed significantly increased L-[^35^S]-methionine incorporation compared with that observed in unstimulated synaptoneurosomes from sham or OVX WT female mice at 4 months of age (WT-Sham-US versus WT-Sham-St, *t* = 5.442*; df* = 4; *p* = 0.003; WT-OVX-US versus WT-OVX-St, *t* = 2.649*; df* = 4; *p* = 0.029) (Fig. 2C). In marked contrast, L-[^35^S]-methionine incorporation was completely absent in KCl stimulated synaptoneurosomes from OVX *APP/PS1* female mice at 4 months of age as compared with that in unstimulated synaptoneurosomes (APP/PS1-Sham-US versus APP/PS1-Sham-St, *t* = 6.62; *df* = 4; *p* = 0.001, APP/PS1-OVX-US versus APP/PS1-OVX-St, *t* = 0.06526*; df* = 4; *p* = 0.476) (Fig. 2C), and these results were comparable to those observed in young *APP/PS1* male mice [45]. Further, stimulation of synaptoneurosomes with KCl from sham *APP/PS1* female mice showed similar levels of L-[^35^S]-methionine incorporation compared with stimulated condition of sham WT female mice (Fig. 2C). Altogether, our results provide evidence that ovariectomy regulates synaptic activity dependent protein translation in the hippocampus of *APP/PS1* female mice.

### Downregulation of synaptosomal Akt1, mTOR phosphorylation and their downstream effectors in aged *APP/PS1* female mice

Akt1-mTOR signaling pathway has emerged as an essential regulator of neuronal survival and activity dependent local dendritic protein translation. At 8 months of age, activity dependent protein translation was impaired in *APP/PS1* female mice and new protein synthesis is mediated by the Akt1-mTOR pathway. Thus, we aimed to understand the molecular mechanisms that underlie the loss of activity dependent protein translation at synapses by investigating the status of phosphorylation of Akt1 and mTOR and their downstream targets in synaptosomes isolated from *APP/PS1* mice.

**Fig. 3:**
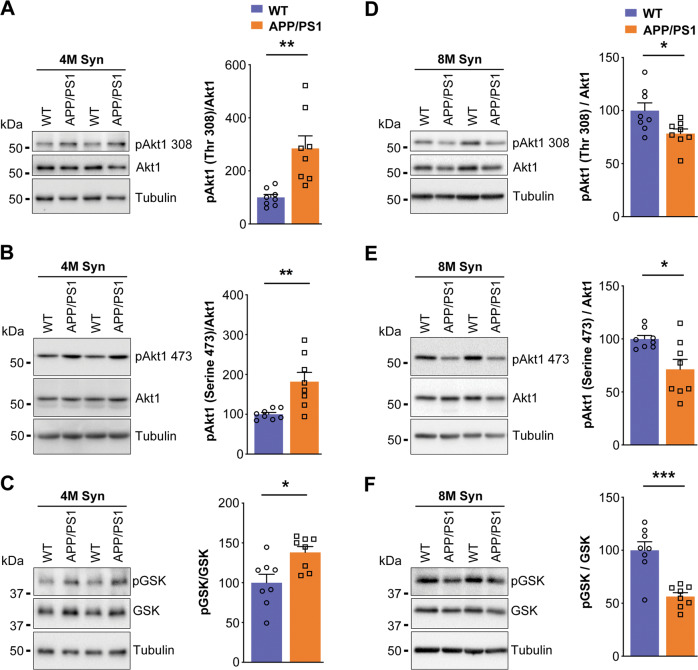
Phosphorylation of Akt1 and GSK3β is increased in synaptosomes of *APP/PS1* female mice at 4 months of age while decreased at 8 months of age. Synaptosomes from 4- and 8- months old WT and *APP/PS1* female mouse brain cortex were subjected to SDS-PAGE followed by western transfer and representative immunoblots were probed with (**A** and **D**) phospho-Akt1 (threonine-308) antibody, (**B** and **E**) phospho-Akt1 (serine-473) antibody, and (**C** and **F**) phospho-GSK3β antibody. Subsequently, these blots were stripped and reprobed, sequentially, for Akt1, GSK3β and tubulin, respectively. (**A**, **B**, **D** and **E**) The data were quantified by measuring phosphorylated Akt1/Akt1 ((**A**) *t* = *3.801; df* = 14; *p* = 0.0019; Hedges’ g (95% CI) 1.79 (0.63, 2.91), (**B**) *t* = *3.479; df* = 14; *p* = 0.0037; Hedges’ g (95% CI) 1.64 (0.51, 2.72), (**D**) *t* = *2.555; df* = 14; *p* = 0.0229; Hedges’ g (95% CI) −1.20 (−2.21, −0.16), (**E**) *t* = *2.877; df* = 14; *p* = 0.0122; Hedges’ g (95% CI) −1.36 (−2.39, −0.28)), (**C** and **F**) phosphorylated-GSK3β/GSK3β ((**C**) *t* = *2.958; df* = 14; *p* = 0.0104; Hedges’ g (95% CI) 1.39 (0.32, 2.43), (**F**) *t* = *4.977; df* = 14; *p* = 0.0002; Hedges’ g (95% CI) –2.35 (−3.59, −1.06)) band intensity ratios and Akt1, GSK3β, and tubulin band intensities were quantified using Bio-Rad image lab software 5.1. Statistical analysis: Two-sided, t-test was used for comparison between WT versus *APP/PS1* mouse groups. Data are presented as mean ± s.e.m. *p < 0.05; **p < 0.01; ***p < 0.001. *n* = 8 mice per group.

We assessed the Akt1-mTOR signaling pathway at 4 months of age, we detected phosphorylation of Akt1 (threonine-308 (*t* = 3.801*; df* = 14; *p* = 0.0019) and serine-473 (*t* = 3.479*; df* = 14; *p* = 0.0037), and GSK3β phosphorylation (*t* = 2.958; *df* = 14; *p* = 0.0104)) was significantly upregulated in synaptosomes of *APP/PS1* female mice compared with that in synaptosomes of WT female mice (Fig. 3A–C).

However, when we assessed at 8 months of age, the phosphorylation of Akt1 at threonine-308 (*t* = 2.555; *df* = 14; *p* = 0.0229) and serine-473 (*t* = 2.877; *df* = 14; *p* = 0.0122) was significantly decreased in synaptosomes compared with WT female mice (Fig. 3D, E). Similarly, phosphorylation of GSK3β (serine-9), a downstream effector molecule in this pathway, was significantly downregulated (*t* = 4.977; *df* = 14; *p* = 0.0002) in synaptosomes of 8 months old *APP/PS1* female mice (Fig. 3F).

**Fig. 4:**
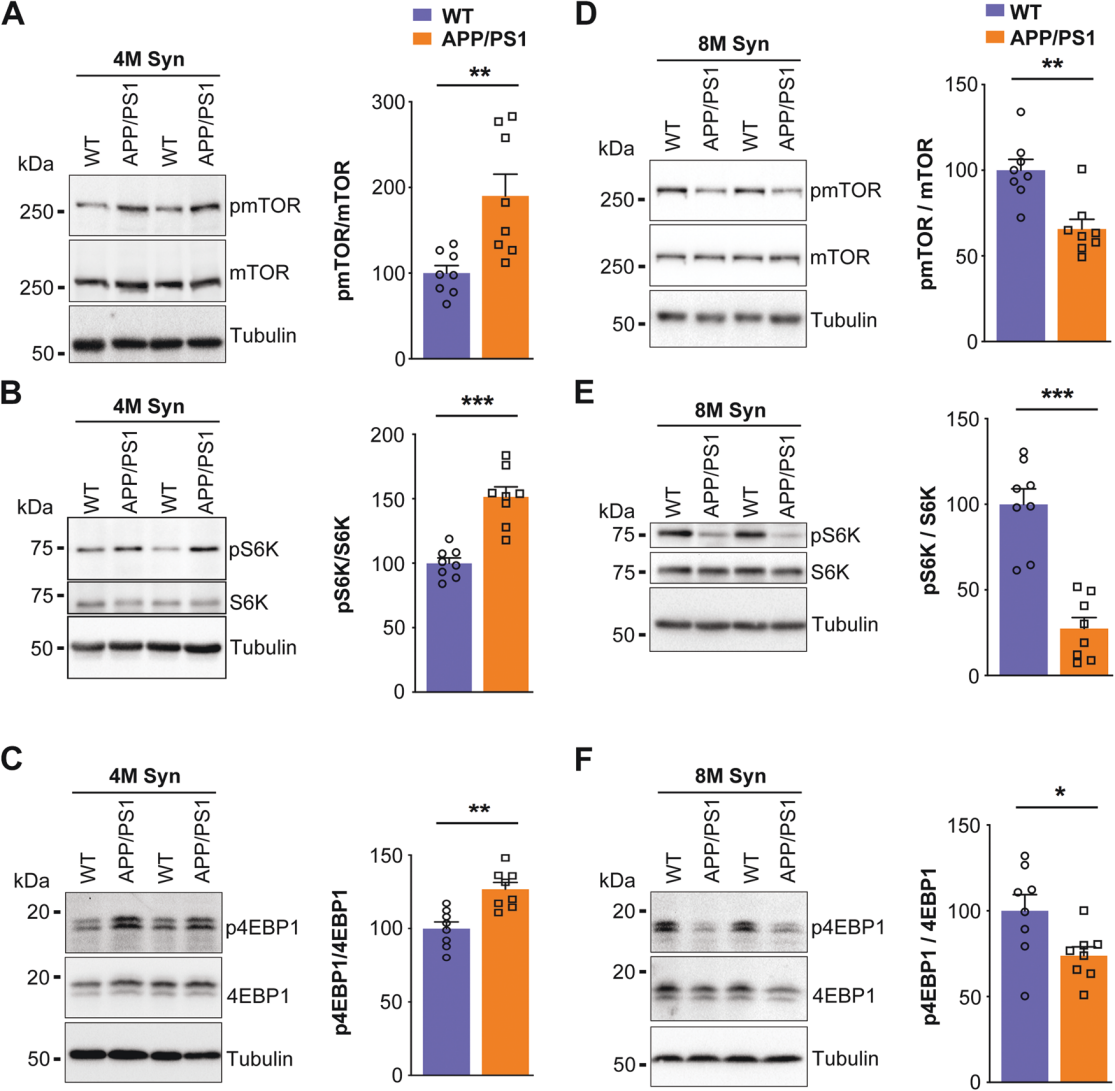
Phosphorylation of mTOR, p70S6K, and 4E-BP1 is increased in synaptosomes of *APP/PS1* female mice at 4 months of age but decreased at 8 months of age. Synaptosomes from the mouse brain cortex of 4 (**A**–**C**) and 8 (**D**–**F**) months old WT and *APP/PS1* female mice were subjected to western blot analysis for (**A** and **D**) phospho-mTOR (serine-2448) antibody, (**B** and **E**) phospho-p70S6K (threonine-389) antibody, and (**C** and **F**) phospho-4E-BP1 (threonine-37/46). Later, these blots were stripped and reprobed, sequentially, for mTOR, p70S6K, 4E-BP1, and tubulin, respectively. **A** and **D** The data were quantified by measuring phosphorylated mTOR/mTOR ((**A**) *t* = *3.367; df* = 14; *p* = 0.0046; Hedges’ g (95% CI) 1.59 (0.47, 2.66), (**D**) *t* = *4.059; df* = 14; *p* = 0.0012; Hedges’ g (95% CI) −1.91 (−3.06, −0.73)), (**B** and **E**) phosphorylated-p70S6K/p70S6K, ((**B**) *t* = *5.908; df* = 14; *p* < 0.0001; Hedges’ g (95% CI) 2.79 (1.38, 4.15), (E) *t* = *6.757; df* = 14; *p* < 0.0001; Hedges’ g (95% CI) −3.19 (−4.66, −1.67)) and (**C** and **F**) phosphorylated-4E-BP1/4E-BP1 ((C) *t* = *4.159; df* = 14; *p* = 0.0010; Hedges’ g (95% CI) 1.96 (0.77, 3.11), (**F**) *t* = *2.426; df* = 14; *p* = 0.0294; Hedges’ g (95% CI) −1.14 (−2.14, −0.112)) band intensity ratios were measured. Statistical analysis: Two-sided *t*-test was conducted to compare between WT versus *APP/PS1* mouse groups. Data are presented as mean ± SEM. *p < 0.05; **p < 0.01; ***p < 0.001. *n* = 8 mice per group.

Mammalian target of rapamycin (mTOR) is a rapamycin sensitive serine/threonine protein kinase and activated by phosphorylation of Akt. Subsequently, activated mTOR stimulates cap-dependent protein synthesis by phosphorylating mRNA translation factors such as eukaryotic initiation factor 4E-binding protein-1 (4E-BP1) and p70 ribosomal S6 kinase (S6K). In this study, phosphorylation of mTOR at serine-2448 (*t* = 3.367*; df* = 14; *p* = 0.0046), phosphorylation of p70S6K (*t* = 5.908; *df* = 14; *p* < 0.0001), and phosphorylation of 4E-BP1 (*t* = 4.159*; df* = 14; *p* = 0.0010) was significantly increased in 4 months old *APP/PS1* female mice (Fig. 4A–C) supporting the fact that the Akt1-mTOR pathway was not compromised in females as seen in males [45].

We observed robust downregulation of mTOR phosphorylation at serine-2448 in synaptosomes of 8 months old *APP/PS1* female mice (*t* = 4.059*;* *df* = 14; *p* = 0.0012) (Fig. 4D). Further, phosphorylation of p70S6K at threonine-389 (*t* = 6.757; *df* = 14; *p* < 0.0001) and phosphorylation of 4E-BP1 (*t* = 2.426; *df* = 14; *p* = 0.0294) was also significantly downregulated in synaptosomes of 8 months old *APP/PS1* female mice as compared with age matched WT female mice (Fig. 4E, F). In conclusion, dysregulation of Akt1-mTOR signaling occurring in synaptosomes of *APP/PS1* female mice at 8 months of age, which was similar to that observed in male mice at 1–3 months of age, could potentially contribute to the inhibition of activity dependent protein translation only at older age in female than in male mice.

### Delayed cognitive decline is observed in women with familial AD mutations

Decline in cognitive performance with increasing age was assessed in men (*n* = 212) and women (*n* = 280) DIAN participants. The participants were grouped as carriers versus non-carriers of familial AD mutations in the genes *APP*, *PSEN1* or *PSEN2*. The characteristics of study participants have been described in Supplementary Table 1. We assessed performance on Wechsler’s logical memory test and word list recall which are tests for episodic memory [49–51], and on the Mini-Mental State Exam (MMSE) [52] using linear mixed effects models.

Differences in performance between men and women participants on the MMSE [52] were evaluated. MMSE score in both mutation carrier (*p* = 0.3819) and non-carrier women participants (*p* = 0.7030) as a function of age showed no statistical significant differences in cognitive decline compared to men participants (Supplementary Figure. 1).

**Fig. 5:**
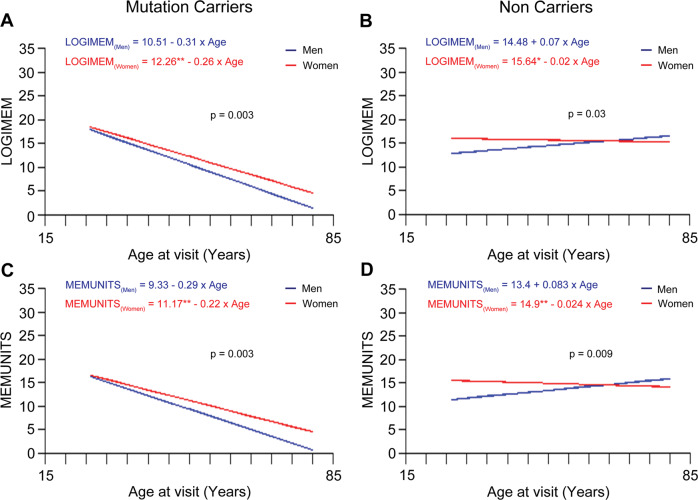
Performance on Wechsler’s logical memory test in men mutation carriers as compared to women mutation carriers. **A** The linear regression trend lines from linear mixed effects models for longitudinal data on Wechsler’s logical memory test score with age (immediate recall, LOGIMEM) from women and men participants in the mutation and non-carriers groups. Performance in the LOGIMEM test was significantly better in women mutation carriers compared to men mutation carriers suggesting that overall performance of women is significantly higher than men irrespective of the age of entry in the study (men equation, *y* = −0.306 × age + 10.51; women equation, *y* = −0.258 × age + 12.266; men vs women, *p* = 0.003). **B** Performance in the LOGIMEM test was also significantly higher in women compared to men in non-carriers, but regression lines are different, suggesting that overall performance of women is statistically significant than men subjects irrespective of the age of entry in the study (men equation, *y* = 0.068 × age + 14.478; women equation, *y* = −0.016 × age + 15.643; men vs women, *p* = 0.03) **C** Performance in the MEMUNITS test was significantly higher in women compared to men mutation carriers suggesting that overall performance of women is significantly higher than men irrespective of the age of entry in the study (men equation, *y* = −0.289 × age + 9.33; women equation, *y* = −0.223 × age + 11.163; men vs women, *p* = 0.003). **D** Intercept of the performance in the MEMUNITS test was significantly higher in women versus men in non-carriers (men equation, *y* = 0.083 × age + 13.392; women equation, *y* = −0.024 × age + 14.899; men vs women, *p* = 0.0091). The *p*-value is for the interaction term of age*sex.

In the Wechsler’s logical memory test, participants are presented with a logically organized story which, they are asked to recall immediately (LOGIMEM) and after a delay of approximately 20 min (MEMUNITS) [50, 53]. Upon performing the linear mixed effects model on the longitudinal data, we observed that among the mutation carriers, men participants showed a significantly greater decline in performance on both immediate (Fig. 5A; *p* = 0.003) and delayed (Fig. 5C; *p* = 0.003) test of logical memory as compared to women participants irrespective of age. Women non-carriers showed slower in cognitive decline compared to men (Fig. 5B; *p* = 0.03) and (Fig. 5D; *p* = 0.0091).

We then evaluated cognitive decline as assessed by word list recall in men and women mutation carriers. Word list recall involved the oral presentation of 16 unrelated words to the participants, at a rate of approximately 1 word per second; then, they were asked to recall the list in any order immediately (WORDIM) and after 20−30 min (WORDDEL). We observed no significant age-related difference in performance of men and women in immediate and delayed word list recall in the mutation carrier (WORDIM; *p* = 0.2414) and (WORDDEL; *p* = 0.211) and non-carrier groups (WORDIM; *p* = 0.5602) and (WORDDEL; *p* = 0.2304). (Supplementary Fig. 2). Altogether, comparison of the slopes obtained from the linear fit showed that sex has influence on the cognitive performance such as Wechsler’s logical memory, in an age dependent fashion, wherein women showed slower decline in all age groups compared to men.

**Fig. 6:**
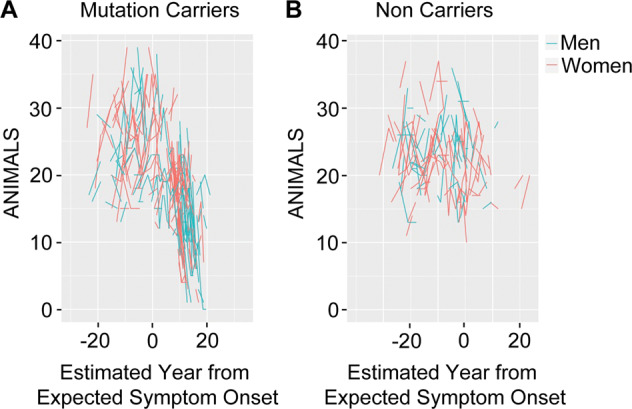
Individual longitudinal performance on category fluency (animals) test (lexical ability and executive control) and measures over estimated year from expected symptom onset (EYO) in men and women mutation carriers and non-carriers. **A**, **B** Spaghetti plot of observed category fluency (Animals) test scores as a function of estimated year from expected symptom onset (EYO) from women (red) and men (cyan) mutation carriers and non-carriers, respectively. (Mutation carriers, men vs women, CDR > 0, ß = −1.14 (SE = 0.44), *p*-value = 0.01; Non-carriers, men vs women, CDR > 0, ß = −0.40 (SE = 0.59), *p*-value = 0.50). The longitudinal data is estimated using the rate of change from the linear mixed effects models over the years (Supplementary Table. 2).

We also analyzed the cognitive performance based on the EYO [47]. As expected, CDR = 0 (cognitively normal) non-carriers perform the best across tests and exhibit the slowest rate of change as individuals approach EYO = 0 (see Supplementary Table. 2 for model results). Symptomatic (CDR > 0) mutation carriers are the individuals who perform the worst and also decline the fastest, although estimates of rate of change did not reach traditional statistical significant thresholds except for MMSE and word immediate recall. Importantly, results from our analyses failed to identify statistically significant sex differences in the absolute scores or rate of change in cognition over time in all groups, (e.g., Wechsler’s logical memory, Supplementary Fig. 3 and Supplementary Table. 2). The one exception is performance on the animal naming test (Fig. 6A, B) (lexical ability and executive control), in this category fluency performance in controlled association the participants are asked to name as many different animals as possible in 1 min, in symptomatic (CDR > 0) mutation carriers where women declined faster than men (ß = −1.14 (SE = 0.44), *p-*value *−*0.01*)* (Fig. 6A).

## Discussion

To understand the molecular underpinnings of the sex difference, we examined a mouse model of AD, *APPswe/PS1ΔE9* mice, which carry both the APP Swedish mutation and a deletion of exon-9 in *PSEN1*. Recent studies from our lab have revealed that male *APP/PS1* mice at 2 months of age exhibit memory impairments following cFC [46, 54]. We examined the ability to recall after cFC in both male and female mice from 2 months to 12 months. The female mice were able to recall after cFC up to 8 months of age, after which there was significant deficit in their ability to recall. In the males, the recall deficits started at 2 months of age and observed until 12 months of age [46]. Studies suggest that estrogen levels in mice are stable until estropause, whereupon at ~8–10 months of age there is a significant reduction in estrogen levels [55–58]. Ovariectomy reduced sex hormone levels [59], impaired learning and memory in rats [60, 61], and in *APP21* rat model [62]. In AD transgenic mouse models, sex and hormone levels affect amyloid load and cognitive functions [36]. To evaluate the potential protective role of estrogen, we analyzed the behavior of ovariectomized WT and *APP/PS1* female mice. The ovariectomized mice showed recall deficits, much as males of the same age (Fig. 2A, B) and our findings are supported with other reports [36, 62].

Learning and memory rely on activity dependent protein translation at the synapse [63, 64], which depends on Akt-mTOR signaling [65, 66]. Activity dependent protein translation and Akt-mTOR signaling are disrupted in 1–9-month-old *APP/PS1* male mice [45]. However, young *APP/PS1* female mice (4 months old) didn’t show activity dependent protein translation deficits until 8 months old (Fig. 1C, D). Female *APP/PS1* mice demonstrated upregulation of the Akt-mTOR pathway in synaptosomes at 4 months, but male mice showed downregulation [45]. However, Akt-mTOR pathway upregulation was not found in middle-aged mice, and by 8 months of age, *APP/PS1* female mice displayed significant downregulation of Akt-mTOR signaling cascade, comparable to males from 1 month onwards [45, 46]. In AD model systems, increased levels of Akt1 or active pAkt1 explain neuroprotection mediated by neurotrophins [67], estrogen [67], and lipoic acid [68], similar with our results. Further, our data show that the dysregulation of Akt1 and mTOR signaling in *APP/PS1* mice may be a factor in behavioral deficits, and its dysregulation is age and sex dependent. Our findings are consistent with other observations that estrogen in ovariectomized mice improves behavioral tasks that dependent on hippocampal memory [69, 70]. Thus, increase in Akt-mTOR signaling at the synapse may be critical for synaptic plasticity throughout life course, particularly in post-estropausal females. In *APP/PS1* mice, we detect a remarkable sex difference in disease development, with male animals showing early behavioral impairment that progresses over time, whereas, in female mice, the impairments did not appear until eight months of age, and thereafter progresses rapidly. Therefore, the progression of behavioral deficits in male and female mice is quite different and can be hypothesized that in male mice, since the deficits starts early, compensatory responses would occur in the brain. However, estrogen is a neuroprotective agent and can counteract the pathogenic effects of β-amyloid accumulation until menopause. However, when estrogen levels decline, there is a substantial difference in the progression of disease.

Numerous studies on sex differences in AD have been limited to clinical diagnoses [71–73]. Some studies revealed a stronger association between AD pathology and clinical AD in women [72, 74], whereas others did not [71, 75, 76]. According to the Framingham Study, a 65-year-old man has a 6.3% lifetime chance of AD and a 10.9% lifetime risk of any dementing disease; while a woman has 12% and 19%, respectively [77]. Estradiol declines following menopause are associated with verbal memory decline [78, 79]. The Colombian Alzheimer’s Prevention Initiative Biomarker Study found no difference in cognitive function (CERAD total score) between men and women *PSEN1* mutation carriers (20-56 years) [80]. *PSEN1/2* mutant carriers have early-onset dementia, but autosomal dominant and late-onset AD have different behavioral and pathophysiology. To evaluate the translational potential to performance in humans with AD, we examined the sex-specific difference in the rate of cognitive decline in the DIAN cohort [22, 47, 81–85]. We found that men mutation carriers had steeper cognitive decline on immediate and delayed test of logical memory as compared to women mutation carriers (Fig. 5A and C). Perhaps many of the observed sex differences due to post-menopausal sex hormone reduction in women is less of a factor in familial AD given the young ages at onset. AD mouse models mirror the human population, in that they are familial AD mutation carriers and females are resistant to AD-related cognitive decline prior to menopause/estropause. Our findings are consistent where women *PSEN1* mutation carriers exhibited better verbal memory than men [80] and the Alzheimer’s Prevention Initiative (API) ADAD Colombia Trial study (30-53 years old) found that women mutation carriers had better delayed recall than men [86, 87]. In women, the average age of menopause onset is between 50−52 years [39, 88]. We find that the rate of change in performance in cognitive tests in mutation carriers showed different trends when the performance was assessed in women versus men as function of age, where men exhibited more rapid cognitive decline than women. We demonstrate that the progression of the disease potentially changes prior to menopause (before 50-52 years) as compared to post-menopause. This is also evident in the sporadic AD, where it has often been shown that women have a rapid cognitive decline in their later years than men [89, 90].

We investigated the intercept and rate of change using linear mixed effects models, taking into consideration clinical dementia rating (CDR) and estimated year from expected symptom onset (EYO) [47]. CDR = 0 non-carriers performed best in all tests and showed the slowest rate of change as individuals approached EYO = 0 (Supplementary Table 2). Symptomatic mutation carriers (CDR > 0) declined the fastest, but estimates of rate of change did not reach statistical significance except for the MMSE test in mutation carriers and word immediate recall in the symptomatic subgroup. In the symptomatic mutation carrier subgroup, women declined faster than men on the animal naming test (lexical ability and executive control) (Fig. 6). The performance on cognitive measures such as immediate and delayed logical memory, analyzed as a function of chronological age, showed that cognitive decline in women was slower as compared to men. Immediate and delayed logical memory tests are not significantly different between men and women when analyzed as a function of EYO. Further, the performances on cognitive tests were not significantly different between men versus women when the analysis was performed in subgroups defined by CDR. Therefore, the results are conflicting and differ depending on whether chronological age, EYO or CDR are considered for assessing the cognitive decline. Nevertheless, our studies suggest potential difference in the trajectory of cognitive decline in men and women, especially when the onset of the disease occurs earlier in life before the age of fifty years.

Our findings indicate that premenopausal women are protected from memory deficits, that hormone replacement therapy may be beneficial, and that the cost-benefit needs to be reevaluated in light of the increasing global burden of AD and the fact that women are disproportionately affected. We conclude that in both humans and mouse models, protection exists so long as estrogen levels are adequate. It protects even in the presence of AD mutations and delays the age of onset. Therefore, estrogen offers substantial benefits and deeper understanding of the signaling pathways is necessary to identify creative approaches to lessen the side effects of hormone replacement treatment.

## Supplementary information

Figure Legends to Supplementary Data

Supplementary Methods

Supplementary Table. 1

Supplementary Table. 2

Supplementary Figure. 1

Supplementary Figure. 2

Supplementary Figure. 3

Supplementary Figure. 4

DIAN Consortia Authors List
