# Supplementary material for: Sex difference in evolution of cognitive decline: studies on mouse model and the Dominantly Inherited Alzheimer Network cohort
Source: Transl Psychiatry. 2023 Apr 12;13:123. doi: 10.1038/s41398-023-02411-8 (PMC10097702; doi:10.1038/s41398-023-02411-8)
Supplement: Supplementary file 1 — Figure Legends to Supplementary Data [file 41398_2023_2411_MOESM1_ESM.docx]

**Sex difference in evolution of cognitive decline: Studies on mouse model and the Dominantly Inherited Alzheimer Network cohort**

Reddy Peera Kommaddi^1*^, Aditi Verma^2^, Graciela Muniz-Terrera^3,4^, Vivek Tiwari^1^, Keerthana Chithanathan^2^, Latha Diwakar^1^, Ruturaj Gowaikar^2^, Smitha Karunakaran^2^, Palash Kumar Malo^1^, Neill R Graff-Radford^5^, Gregory S Day^5^, Christoph Laske^6,7^, Jonathan Vöglein^8, 9^, Georg Nübling^8,9^, Takeshi Ikeuchi^10^, Kensaku Kasuga^10^, the Dominantly Inherited Alzheimer Network (DIAN)^11, #^ and Vijayalakshmi Ravindranath^1,2^

**FIGURE LEGENDS TO SUPPLEMENTARY DATA**

**Supplementary Figure 1.**

**Linear regression of MMSE scores with age for men and women mutation carriers and non-carriers**. (A-B) The linear regression trend lines from linear mixed effects models for longitudinal data on MMSE test score with age from women and men participants in the mutation and non-carrier groups. MMSE performance demonstrated no significant sex differences in the mutation carrier (men equation, y = -0.324 x AGE + 26.072; women equation, y = -0.265 x AGE + 26.583; men vs women p=0.3819) and non-carrier groups (men equation, y = 0.018 x AGE + 29.094; women equation, y = -0.005 x AGE + 29.152; men vs women p=0.7030). The p-value is for the interaction term of age*sex. Rate of decline in women mutation carriers is shown in red line and men mutation carriers is shown in blue line.

**Supplementary Figure 2.**

**Performance on word list recall test in men and women mutation carriers and non- carriers.**

(A-D) The linear regression trend lines from linear mixed effects models for longitudinal data of the word list recall test (immediate recall, WORDIM and delayed recall, WORDDEL) scores with age for men and women mutation carrier (WORDIM, men equation, y = -0.134 x AGE + 4.486; women equation, y = -0.119 x AGE + 4.773, men vs women p=0.2414; WORDDEL, men equation, y = -0.111 x AGE + 1.861; women equation, y = -0.104 x AGE + 2.108, men vs women p=0.2110) and non-carrier groups (WORDIM, men equation, -0.029 x AGE + 5.787; women equation, y = -0.03 x AGE + 5.936, men vs women p=0.5602; WORDDEL, men equation, y = -0.044 x AGE + 2.951; women equation, y = -0.048 x AGE + 3.278, men vs women p=0.2304). The p-value is for the interaction term of age*sex. Red line indicates women and blue line indicates men.

**Supplementary Figure 3.**

**Performance on Wechsler’s logical memory test in men and women mutation carriers and non- carriers as a function of baseline clinical dementia rating (CDR) score.** (A-B) Model estimated mean trajectory form linear mixed effects models for longitudinal data on Wechsler’s logical memory test score (immediate recall, LOGIMEM) as a function of estimated year from expected symptom onset (EYO) in women and men mutation carrier and non-carrier groups. (Mutation carriers, men vs women, CDR>0, ß=0.12 (SE=0.15), p=-0.43; Non-carriers, men vs women, CDR>0, ß=1.34 (SE=0.72), p=0.08). (C-D) Model estimated mean trajectory from longitudinal data on delayed recall (MEMUNITS) as a function of estimated year from expected symptom onset (EYO) in women and men mutation carrier and non-carrier groups. (Mutation carriers, men vs women, CDR>0, ß=-0.41 (SE=0.80), p=0.60; Non-carriers, men vs women, CDR>0, ß=0.92 (SE=0.49), p=0.08). The average EYO at study entry is ~8 years (-8.75 yrs).

**Supplementary Figure 4:**

**Amytacker stains Aβ plaques in APP/PS1 mice brains.**

Coronal brain sections depicting cortical regions from Wild type and APP/PS1 (male and female, 10 months of age) mice were stained with Amytracker 520. Extracellular deposits of β-amyloid peptide observed as positively stained plaques (green fluorescent) in APP/PS1 mice. Scale bar = 50 μm
