# Supplementary material for: Sex difference in evolution of cognitive decline: studies on mouse model and the Dominantly Inherited Alzheimer Network cohort
Source: Transl Psychiatry. 2023 Apr 12;13:123. doi: 10.1038/s41398-023-02411-8 (PMC10097702; doi:10.1038/s41398-023-02411-8)
Supplement: Supplementary file 2 — Supplementary Methods [file 41398_2023_2411_MOESM2_ESM.docx]

**Sex difference in evolution of cognitive decline: Studies on mouse model and the Dominantly Inherited Alzheimer Network cohort**

Reddy Peera Kommaddi^1*^, Aditi Verma^2^, Graciela Muniz-Terrera^3,4^, Vivek Tiwari^1^, Keerthana Chithanathan^2^, Latha Diwakar^1^, Ruturaj Gowaikar^2^, Smitha Karunakaran^2^, Palash Kumar Malo^1^, Neill R Graff-Radford^5^, Gregory S Day^5^, Christoph Laske^6,7^, Jonathan Vöglein^8, 9^, Georg Nübling^8,9^, Takeshi Ikeuchi^10^, Kensaku Kasuga^10^, the Dominantly Inherited Alzheimer Network (DIAN)^11, #^ and Vijayalakshmi Ravindranath^1,2^

**Supplementary methods:**

**Contextual fear conditioning.** Contextual fear conditioning (cFC) behavior was assessed as reported previously [51]. We utilized a fear conditioning chamber that could deliver a foot shock from a bottom steel grid floor (Coulbourn Instruments, Whitehall, PA, USA). The fear conditioning chamber and bottom grid floor were wiped with 70% ethanol before and after each experiment. The identity of the context was maintained with the presence of distinct odor (2% acetic acid [vol/vol]). Mice were group housed and were handled prior to conditioning for habituation for five min for three days. On the contextual fear conditioning day, mice were placed in the conditioning chamber and allowed to explore the training context for 1 min, and then given three foot shocks, (2 s and 0.6 mA each, inter-trial interval: 30 s) and allowed to stay in the camber for an additional 30 s. Following conditioning, all mice were returned to their home cages. Long-term fear memory for the context was evaluated the following day (day 2; ~24 hr following condition). To evaluate long term fear memory to the context, mice were again placed in the conditioning chamber and allowed to explore for 1 min, after which freezing to the context was assessed for further 90 s without any foot shock to evaluate contextual memory retrieval and freezing response was analyzed.

**Preparation of synaptosomes.** Synaptosomal fractions were isolated as described previously [47]. Briefly, mouse cortical brain tissue was minced in a small volume of homogenization buffer (5mM HEPES buffer, pH 7.4, containing 0.32 M sucrose, 50 mM sodium fluoride, 1 mM sodium orthovanadate, 2 µg/ml aprotinin, 10 µg/ml leupeptin, 7 µg/ml pepstatin A, 100 µg/ml of phenylmethanesulfonyl fluoride [PMSF] and 10 µl/ml of protease inhibitor cocktail) and the minced tissue was transferred to a cold Potter**-**Elvehjem homogenizer and homogenized in 10 volumes of homogenization buffer using electronic motor controlled homogenizer (Glas-Col, Terre Haute, IN, USA). The homogenate was transferred to pre-cooled centrifuge tubes and centrifuged at 1000× g for 10 min at 4°C. The post-nuclear supernatant (PNS) was collected from each tube and transferred to new pre-cooled centrifuge tubes. PNS was centrifuged at 12000× g for 15 min at 4°C, and the obtained pellet was resuspended in 5 mM Tris (pH 8.1) containing 0.32 M sucrose supplemented with protease and phosphatase inhibitors. The discontinuous sucrose gradient tubes (0.85-1.0-1.2 M) were prepared and the resuspended pellet (after centrifugation at 12000× g) was layered on top of the 0.85M sucrose gradient. Discontinuous sucrose density gradient centrifugation was performed in a tabletop ultracentrifuge (Optima MAX-XP ultracentrifuge-Beckman-Coulter, USA) at 85,000× g for 2 hr at 4°C. Synaptosomal fraction obtained at the interface of 1M and 1.2 M sucrose gradient was collected and washed twice in 5 mM Tris (pH 8.1) and resuspended in homogenization buffer for further analysis.

**Immunoblotting.** Equal amounts of synaptosomes were resolved on sodium dodecyl sulfate polyacrylamide gel electrophoresis and transferred to a polyvinylidene difluoride membrane by electroblotting. Immunoblots were blocked in 5% bovine serum albumin for 1 hour at room temperature and immunoblotted with respective primary antibodies and incubated at 4°C overnight. The following day, all immunoblots were washed and incubated at room temperature in respective secondary antibodies. Immunoreactive bands were detected using enhanced chemiluminescence (Clarity Western ECL blotting substrate, Bio-Rad). Immunoreactive signals were acquired using the Bio-Rad Chemidoc-XRS and analysed with Imagelab software (Bio-Rad).

**Isolation of synaptoneurosomes and L-[^35^S]-methionine incorporation assay.** Isolation of synaptoneurosomes from mouse hippocampal tissue and L-[^35^S]-methionine incorporation assay were performed as described previously [47]. Briefly, hippocampal tissue was homogenized with 10 volumes of translation buffer (118 mM NaCl, 4.7 mM KCl, 1.2 mM MgSO_4_, 2.5 mM CaCl_2_, 1.53 mM KH_2_PO_4_, 212.7 mM glucose, and 1 mM DTT), and supplemented with protease and phosphatase inhibitors, 200 μg/ml chloramphenicol and 30 U/ml RNAse inhibitor. The homogenate was then passed sequentially through two 100 μm and one 10 μm nylon mesh filters (Millipore). The filtrate obtained was then centrifuged at 1500×g at 4°C for 10 min and the pellet containing synaptoneurosomes was re-suspended in translation buffer. Stimulation of synaptoneurosomes was carried out by incubation with 50 mM KCl at 37°C for 15 min in presence of 50 μCi L-[^35^S]-methionine. Unstimulated samples were incubated with 50 μCi L-[^35^S]-methionine alone. Samples were then precipitated with equal volume of ice cold 10% (w/v) trichloroacetic acid (TCA). Protein pellets were washed extensively with ice cold 5% (w/v) TCA followed by washes with ice-cold methanol until the washes showed no detectable radioactivity. The washed pellets were resuspended in 0.1 N NaOH and radioactivity was measured using liquid scintillation counter.

**Amytracker staining.** Wild type and APP/PS1 mouse brain sections (30 μm thick) were cut using Cryotome (Leica Biosystems Inc., Buffalo Grove, IL, USA). Sections were fixed with ice-cold ethanol at room temperature for 5 min. Tissue sections were rehydrated for 5 min in a 1:1 mixure of ethanol and water. The sections were then washed with phosphate buffered saline (10 mM, pH 7.4, PBS) for 5 min. The sections were incubated for 30 min with amytracker (1:1000) in PBS. After incubation, the sections were washed with PBS and mounted with Vectashield mounting medium without DAPI (H-1000). Image acquisition was performed using 10x/0.3 (plan neufluar), Zeiss Axio Imager.M2 (Carl Zeiss Microscopy, LLC, Thornwood, NY, USA).
