# Supplementary material for: Sex difference in evolution of cognitive decline: studies on mouse model and the Dominantly Inherited Alzheimer Network cohort
Source: Transl Psychiatry. 2023 Apr 12;13:123. doi: 10.1038/s41398-023-02411-8 (PMC10097702; doi:10.1038/s41398-023-02411-8)
Supplement: Supplementary file 3 — Supplementary Table. 1 [file 41398_2023_2411_MOESM3_ESM.docx]

Table 1. Description of DIAN study participants at first visit.

|  | Women (n=280) | Men (n=212) |
| --- | --- | --- |
| Age, y, mean (SD) | 38.01 (11.2) | 38.05 (10.7) |
| Mutation Carriers, n (%) | 163 (58.2) | 132 (62.3) |
| Non-carriers, n (%) | 117 (41.8) | 80 (37.7) |
| Education, y, mean (SD) | 14.45 (2.7) | 14.43 (3.3) |
| CDR≥0.5, n (%) | 69 (24.6) | 59 (27.8) |
| CDR 0, n (%) | 211 (75.7) | 153 (72.2) |
| AO, y, mean (SD) | 47.04 (7.4) | 47.47 (7.6) |
| APOE-ε4 carrier, n (%) | 80 (28.6) | 69 (32.5) |

y: years; CDR: Clinical dementia rating [91]; AO: A combination of individual age-at-symptomatic onset (symptomatic), mean mutation age-at-symptomatic onset, and parental age-at-symptomatic onset; SD: standard deviation.

91. Morris JC. The Clinical Dementia Rating (CDR): current version and scoring rules. Neurology. 1993;43:2412-2414.
