# Supplementary material for: Sex difference in evolution of cognitive decline: studies on mouse model and the Dominantly Inherited Alzheimer Network cohort
Source: Transl Psychiatry. 2023 Apr 12;13:123. doi: 10.1038/s41398-023-02411-8 (PMC10097702; doi:10.1038/s41398-023-02411-8)
Supplement: Supplementary file 4 — Supplementary Table. 2 [file 41398_2023_2411_MOESM4_ESM.docx]

Table. 2. Cognitive performance in mean intercept and annual rates of change in Mutation Carriers and Non Carriers.

|  |  | Mutation Carriers | | | | Non Carriers | | | |
| --- | --- | --- | --- | --- | --- | --- | --- | --- | --- |
| Cognitive test |  | CDR0 | | CDR>0 | | CDR0 | | CDR>0 | |
|  |  | ß(SE) | p-value | ß(SE) | p-value | ß(SE) | p-value | ß(SE) | p-value |
| MMSE | **Intercept** | 29.04(-.13) | <0.001 | 34.96(2.30) | <0.001 | 29.85(0.43) | <0.001 | 28.16(0.68) | <0.001 |
|  | Age | 0.01(0.01) | 0.38 | **0.36(0.13)** | **0.01** | 0.01(0.03) | 0.71 | 0.10(0.07) | 0.17 |
|  | Female Sex | -0.25(0.19) | 0.20 | -2.96(3.55) | 0.40 | -0.57(0.67) | 0.39 | 2.19(1.39) | 0.36 |
|  | **Rate of change** | -0.04(0.01) | <0.001 | -1.03(0.22) | <000 | 0.008(0.03) | 0.82 | -0.06(0.07) | 0.52 |
|  | Age | -0.001(0.001) | 0.29 | **-0.03(0.01)** | **0.005** | -0.002(0.002) | 0.30 | -0.003(0.007) | 0.71 |
|  | Female Sex | 0.009(0.01) | 0.61 | 0.21(0.32) | 0.50 | -0.007(0.05) | 0.88 | -0.01(0.16) | 0.94 |
| FluF | **Intercept** | 14.58(0.68) | <0.001 | 16.19(5.12) | 0.001 | 13.60(0.47) | <0.001 | 13.63(1.87) | <.001 |
|  | Age | -0.0((0.06) | 0.10 | -0.27(0.36) | 0.44 | 0.007(0.04) | 0.88 | 0.15(0.20) | 0.47 |
|  | Female Sex | -0.19(0.91) | 0.83 | 6.56(7.47) | 0.38 | 0.04(0.63) | 0.94 | -1.97(3.78) | 0.60 |
|  | **Rate of change** | 0.03(0.09) | 0.72 | -0.51(0.39) | 0.19 | 0.001(0.004) | 0.97 | -0.33(0.21) | 0.26 |
|  | Age | -0.003(0.006) | 0.56 | 0.02(0.02) | 0.39 | -0.002(0.002) | 0.39 | 0.002(0.02) | 0.92 |
|  | Female Sex | -0.02(0.12) | 0.85 | -0.48(0.54) | 0.37 | 0.04(0.05) | 0.40 | 0.22(0.46) | 0.64 |
| MEMUNITS | **Intercept** | 13.87)0.59) | <0.00 | 5.05(5.76) | 0.38 | 14.72(0.44) | <0.00 | 14.24(2.64) | <0.001 |
|  | Age | -0.05(0.05) | 0.30 | -0.32(0.37) | 0.38 | -0.05(0.04) | 0.24 | -0.04(0.18) | 0.83 |
|  | Female Sex | -0.46(0.88) | 0.60 | 3.86(8.96( | 0.66 | -1.25(0.58) | 0.03 | -6.07(4.63) | 0.20 |
|  | **Rate of change** | -0.09(0.07) | 0.21 | 0.25(0.55) | 0.64 | 0.008(0.04) | 0.84 | -0.34(0.25) | 0.30 |
|  | Age | -0.002(0.004) | 0.52 | 0.02(0.03) | 0.53 | 0.002(0.001) | 0.27 | 0.03(0.02) | 0.19 |
|  | Female Sex | 0.12(0.08) | 0.14 | -0.41(0.80) | 0.60 | 0.07(0.05) | 0.14 | 0.92(0.49) | 0.08 |
| FLUA | **Intercept** | 11.95(0.71) | <0.0001 | 9.37(3.36) | 0.006 | 11.74(0.41) | <0.001 | 12.72(1.63) | <0.001 |
|  | Age | -0.05(0.06) | 0.38 | -0.07(0.21) | 0.75 | 0.02(0.04) | 0.49 | 0.12(0.17) | 0.48 |
|  | Female Sex | 0.93(0.97) | 0.34 | 2.42(5.16) | 0.63 | 0.63(0.54) | 0.24 | -4.29(3.25) | 0.20 |
|  | **Rate of change** | 0.003(0.08) | 0.96 | -0.10(0.34) | 0.74 | -0.03(0.04) | 0.45 | -0.41(0.20) | 0.17 |
|  | Age | 0.001(0.005) | 0.71 | 0.009(0.02) | 0.65 | 0.002(0.001) | 0.87 | 0.003(0.02) | 0.87 |
|  | Female Sex | -0.02(0.11) | 0.81 | -0.12(0.49) | 0.80 | 0.05(0.05) | 0.31 | 0.48(0.40) | 0.25 |
| FLUS | **Intercept** | 16.14(0.73) | <0.001 | 15.22(3.42) | <0.00 | 15.36(0.45) | <0.001 | 15.29(1.40) | <.001 |
|  | Age | -0.10(0.06) | 0.10 | -0.03(0.22) | 0.86 | 0.04(0.04) | 0.36 | 0.38(0.16) | 0.03 |
|  | Female Sex | -0.31(1.00) | 0.75 | 1.61(5.25) | 0.75 | -0.11(0.60) | 0.85 | 2.69(2.70) | 0.33 |
|  | **Rate of change** | 0.03(0.08) | 0.66 | -0.39(0.34) | 0.26 | -0.05(0.04) | 0.27 | -0.39(0.17) | 0.25 |
|  | Age | -0.005(0.005) | 0.29 | 0.007(0.02) | 0.71 | -0.002(0.002) | 0.24 | -0.02(0.02) | 0.40 |
|  | Female Sex | 0.02(0.11) | 0.82 | -0.07(0.49) | 0.87 | 0.04(0.05) | 0.40 | -0.20(0.37) | 0.62 |
| WORDIM | **Intercept** | 5.84(0.47) | <0.001 | 9.77(2.45) | <0.001 | 5.94(0.19) | <0.001 | 5.29(0.70) | <.001 |
|  | Age | -0.05(0.04) | 0.15 | **-0.35(0.17)** | **0.03** | -0.02(0.02) | 0.18 | -0.05(0.07) | 0.47 |
|  | Female Sex | -0.70)0.64) | 0.27 | -1.73(3.43) | 0.61 | -0.13(0.26) | 0.62 | -1.28(1.40) | 0.37 |
|  | **Rate of change** | -0.12(0.08) | 0.11 | -0.65(0.24) | 0.009 | -0.0009(0.02) | 0.72 | -0.12(0.08) | 0.24 |
|  | Age | 0.006(0.005) | 0.27 | **-0.03(0.02)** | **0.04** | 0.003(0.0009) | 0.96 | 0.003(0007) | 0.70 |
|  | Female Sex | 0.03(0.10) | 0.79 | 0.18(0.34) | 0.60 | 0.003(0.02) | 0.88 | 0.11(0.16) | 0.50 |
| WORDDEL | **Intercept** | 4.16(1.17) | <0.005 | -5.42(5.81) | 0.35 | 4.44(0.61) | <0.001 | 2.80(0.63) | 0.005 |
|  | Age | 0.08(0.11) | 0.48 | -0.67)0.38) | 0.07 | -0.07(0.06) | 0.28 | -0.04(0.06) | 0.52 |
|  | Female Sex | -0.78(1.69) | 0.64 | 3.57(8.92) | 0.68 | -1.17(0.84) | 0.16 | -1.01(1.22) | 0.42 |
|  | **Rate of change** | -0.12(0.14) | 0.37 | 0.66(0.58) | 0.25 | 0.03(0.07) | 0.58 | -0.05(0.07) | 0.53 |
|  | Age | 0.002(0.008) | 0.78 | 0.06(0.03) | 0.07 | -0.003(0.003) | 0.31 | -0.003(0.006) | 0.66 |
|  | Female Sex | -0.11(0.17) | 0.51 | -0.22(0.84) | 0.79 | -0.02(0.07) | 0.80 | -0.02(0.14) | 0.86 |
| LOGIMEM | **Intercept** | 14.61(0.42) | <0.001 | 16.30(1.20) | <0.001 | 15.35(0.40) | 0<0.001 | 17.91 (3.93) | <0.001 |
|  | Age | -0.08(0.04) | 0.03 | 0.13(0.07) | 0.07 | -0.04(0.04) | 0.28 | -0.26(0.26) | 0.34 |
|  | Female Sex | -0.98(0.63) | 0.12 | -2.82(1.91) | 0.14 | -0.80(0.54) | 0.13 | -11.45(7.36) | 0.14 |
|  | **Rate of change** | -0.02(0.05) | 0.63 | -0.79(0.10) | <0.00 | 0.005(0.04) | 0.89 | -0.63(0.35) | 0.21 |
|  | Age | -0.0002(0.003) | 0.94 | -0.01(0.005) | 0.04 | 0.002(0.001) | 0.14 | 0.06(0.02) | 0.12 |
|  | Female Sex | 0.06(0.06) | 0.26 | 0.12(0.15) | 0.43 | 0.06(0.05) | 0.17 | 1.34(0.72) | 0.08 |
| DIGIF | **Intercept** | 8.71(0.20) | <0.001 | 9.92(2.13) | <0.001 | 9.30(0.19) | <0.001 | 8.07(0.84) | <.001 |
|  | Age | 0.007(0.02) | 0.70 | -0.12(0.14) | 0.38 | 0.03(0.02) | 0.12 | 0.14(0.09) | 0.17 |
|  | Female Sex | 0.46(0.30) | 0.12 | 3.55(3.04) | 0.24 | -0.24(0.26) | 0.33 | 1.67(1.73) | 0.35 |
|  | **Rate of change** | -0.01(0.02) | 0.58 | -0.24(0.23) | 0.30 | -0.02(0.01) | 0.20 | -0.03(0.11) | 0.78 |
|  | Age | 0.004(0.001) | 0.75 | 0.02(0.02) | 0.23 | -0.0009(0.0009) | 0.31 | -0.01(0.01) | 0.42 |
|  | Female Sex | 0.02(0.03) | 0.39 | -0.43(0.33) | 0.20 | 0.0002(0.02) | 0.99 | -0.20(0.22) | 0.37 |
| DIGIFLEN | **Intercept** | 6.90(0.09) | <0.001 | 6.44(2.00) | <0.001 | 7.08(0.09) | <0.001 | 6.57(0.48) | <.001 |
|  | Age | 0.01(0.009) | 0.29 | -0.14(0.13) | 0.29 | 0.02(0.009) | 0.07 | 0.06(0.06) | 0.27 |
|  | Female Sex | 0.17(0.14) | 0.23 | 3.71(2.82) | 0.19 | -0.06(0.12) | 0.60 | 0.79(1.01) | 0.44 |
|  | **Rate of change** | -0.01(0.01) | 0.21 | -0.02(0.22) | 0.93 | -0.009(0.01) | 0.35 | -0.05(0.06) | 0.47 |
|  | Age | 0.000(0.000) | 0.97 | 0.02(0.01) | 0.23 | -0.0004(0.0004) | 0.30 | -0.002(0.006) | 0.70 |
|  | Female Sex | 0.02(0.01) | 0.21 | -0.40(0.31) | 0.19 | -0.008(0.01) | 0.46 | -0.004(0.13) | 0.73 |
| DIGIB | **Intercept** | 7.42(0.22) | <0.001 | 3.73(6.40) | 0.55 | 7.63(0.59) | <.001 | 6.94(1.31) | <0.001 |
|  | Age | -0.003(0.02) | 0.85 | -0.41(0.43) | 0.34 | 0.003(0.04) | 0.94 | -0.18(0.09) | 0.06 |
|  | Female Sex | 0.13(0.32) | 0.68 | 10.69(9.57) | 0.26 | 0.62(0.89) | 0.48 | -3.26(2.25) | 0.16 |
|  | **Rate of change** | -0.02(0.03) | 0.35 | 0.90(0.62) | 0.14 | -0.003(0.05) | 0.94 | -0.09(0.09) | 0.42 |
|  | Age | -0.004(0.001) | 0.009 | 0.04(0.04) | 0.27 | -0.001(0.002) | 0.57 | 0.02(0.004) | 0.03 |
|  | Female Sex | 0.02(0.03) | 0.55 | -1.13(0.89) | 0.20 | 0.005(0.006) | 0.93 | 0.38(0.20) | 0.08 |
| ANIMALS | **Intercept** | 22.96(0.68) | <0.001 | 21.95(2.97) | <0.001 | 23.17(0.51) | <0.001 | 20.98(2.90) | <.001 |
|  | Age | -0.14(0.06) | 0.02 | -0.03(0.20) | 0.88 | -0.09(0.05) | 0.05 | 0.07(0.25) | 0.80 |
|  | Female Sex | 0.61(1.01) | 0.54 | **12.31(4.42)** | **0.006** | 0.001(0.69) | 0.99 | 0.84(5.19) | 0.87 |
|  | **Rate of change** | -0.14(0.08) | 0.10 | -0.51(0.31) | 0.10 | 0.02(0.05) | 0.73 | -0.12(0.31) | 0.72 |
|  | Age | -0.01(0.005) | 0.02 | 0.002(0.02) | 0.90 | -0.004(0.002) | 0.03 | -0.02(0.02) | 0.61 |
|  | Female Sex | 0.09(0.10) | 0.35 | **-1.14(0.44)** | **0.01** | 0.01(0.06) | 0.82 | -0.40(0.59) | 0.50 |
| VEG | **Intercept** | 16.14(0.78) | <0.001 | 13.28(4.59) | 0.004 | 16.44(0.37) | <0.001 | 14.64(1.43) | <.001 |
|  | Age | -0.01(0.07) | 0.81 | -0.47(0.30) | 0.12 | -0.002(0.04) | 0.94 | 0.25(0.16) | 0.13 |
|  | Female Sex | -1.80(1.04) | 0.08 | 0.79(6.92) | 0.90 | -1.78(0.50) | 0.0005 | -0.30(2.93) | 0.92 |
|  | **Rate of change** | -0.08(0.10) | 0.42 | -0.20(0.43) | 0.63 | 0.02(0.001) | 0.61 | 0.07(0.17) | 0.72 |
|  | Age | -0.0006(0.007) | 0.92 | 0.04(0.02) | 0.13 | -0.003(0.001) | 0.06 | -0.02(0.02) | 0.58 |
|  | Female Sex | -0.11(0.14) | 0.40 | -0.34(0.62) | 0.58 | 0.01(0.04) | 0.78 | -0.31(0.37) | 0.40 |

The data was coded such as men=1 and women=0, therefore, the coefficient represents the estimated value for women.

MMSE: MINI MENTAL STATE EXAM; FLUF: LETTER FLUENCY LETTER F; FLUA: ETTER FLUENCY LETTER A; FLUS: LETTER FLUENCY LETTER S; WORDIM: WORD LIST RECALL – Immediate; WORDDEL: WORD LIST RECALL – Delayed; MEMUNITS: Wechsler’s Logical Memory Delayed; LOGIMEM: Wechsler’s Logical Memory-Immediate; DIGIF: Digit span forward; DIGIFLEN: Digit span forward length; DIGIB: Digit span backward; DIGIBLEN: Digit span backward length; VEG: Vegetables.
