# Supplementary figures and images for: Sex difference in evolution of cognitive decline: studies on mouse model and the Dominantly Inherited Alzheimer Network cohort
Source: Transl Psychiatry. 2023 Apr 12;13:123. doi: 10.1038/s41398-023-02411-8 (PMC10097702; doi:10.1038/s41398-023-02411-8)

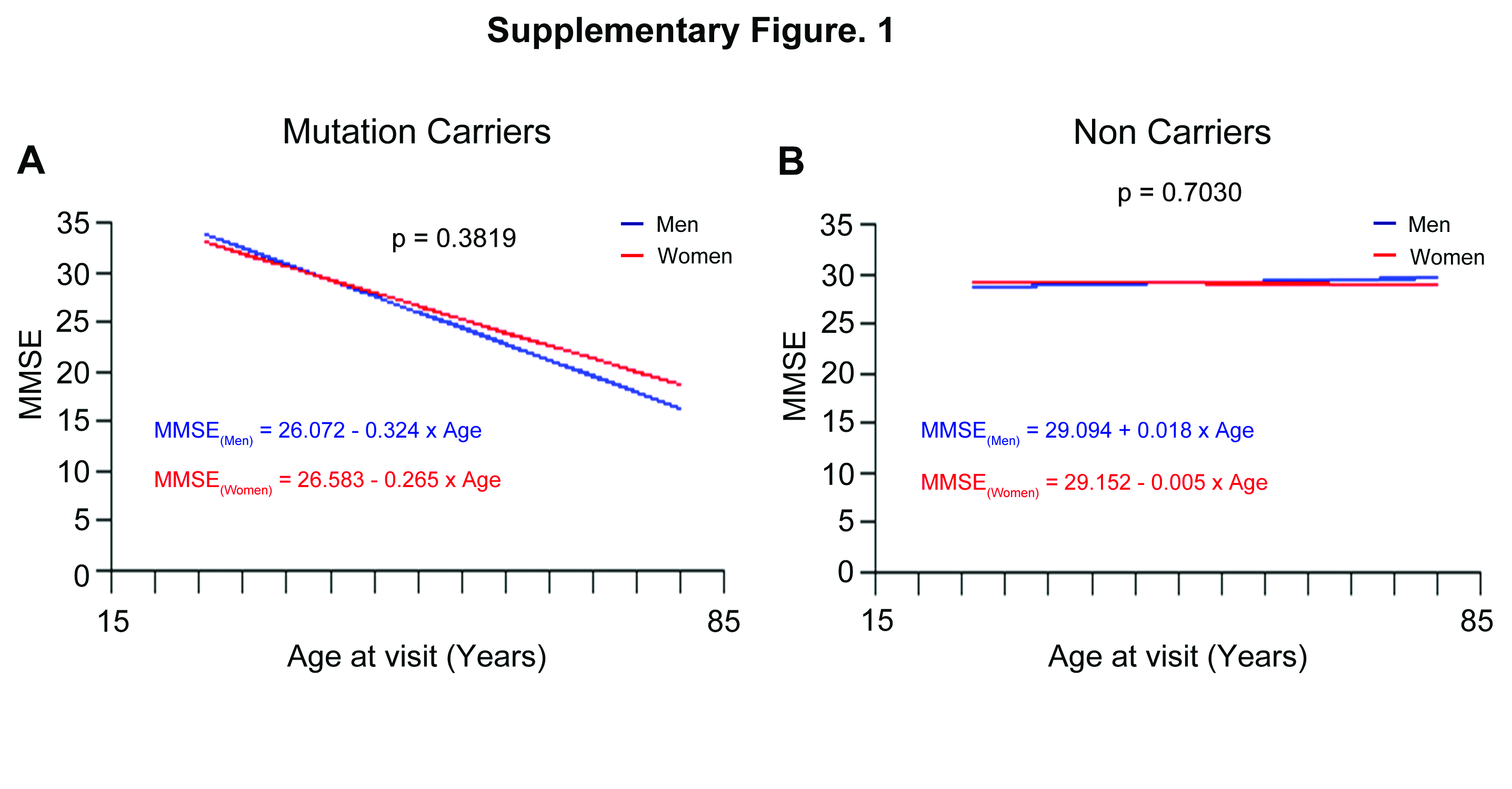

Supplement: Supplementary file 5 — Supplementary Figure. 1 [file 41398_2023_2411_MOESM5_ESM.tif]

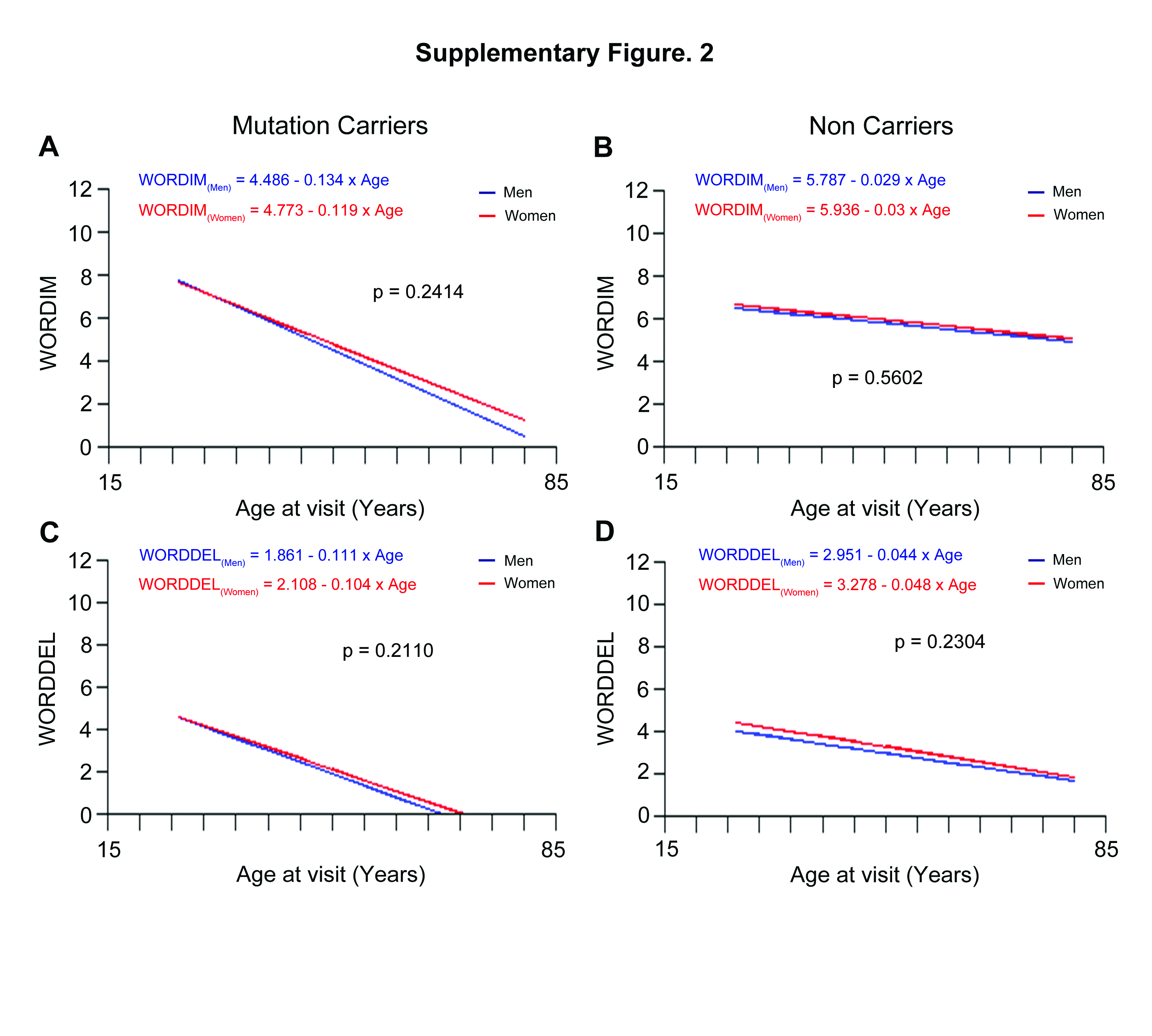

Supplement: Supplementary file 6 — Supplementary Figure. 2 [file 41398_2023_2411_MOESM6_ESM.tif]

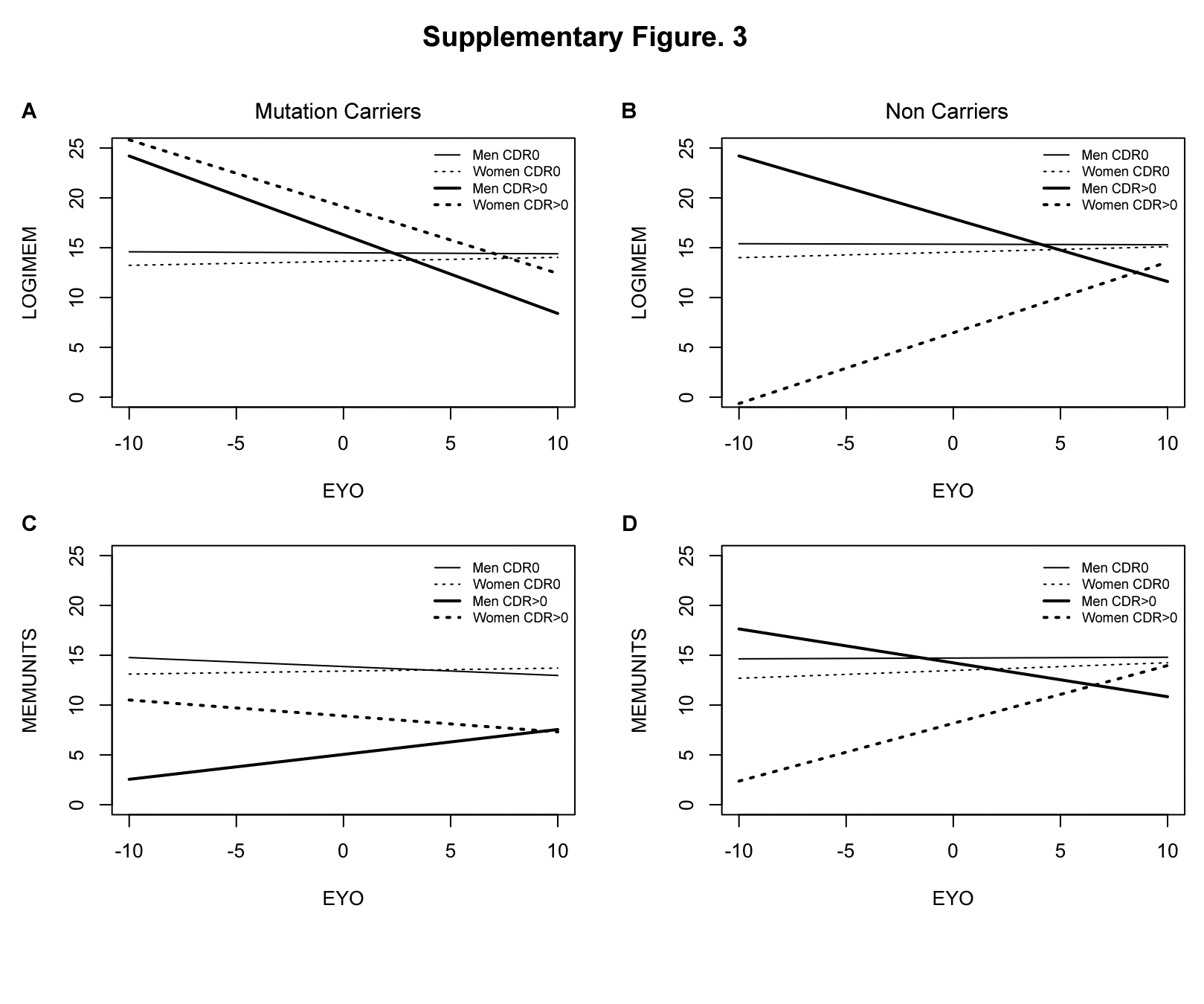

Supplement: Supplementary file 7 — Supplementary Figure. 3 [file 41398_2023_2411_MOESM7_ESM.tif]

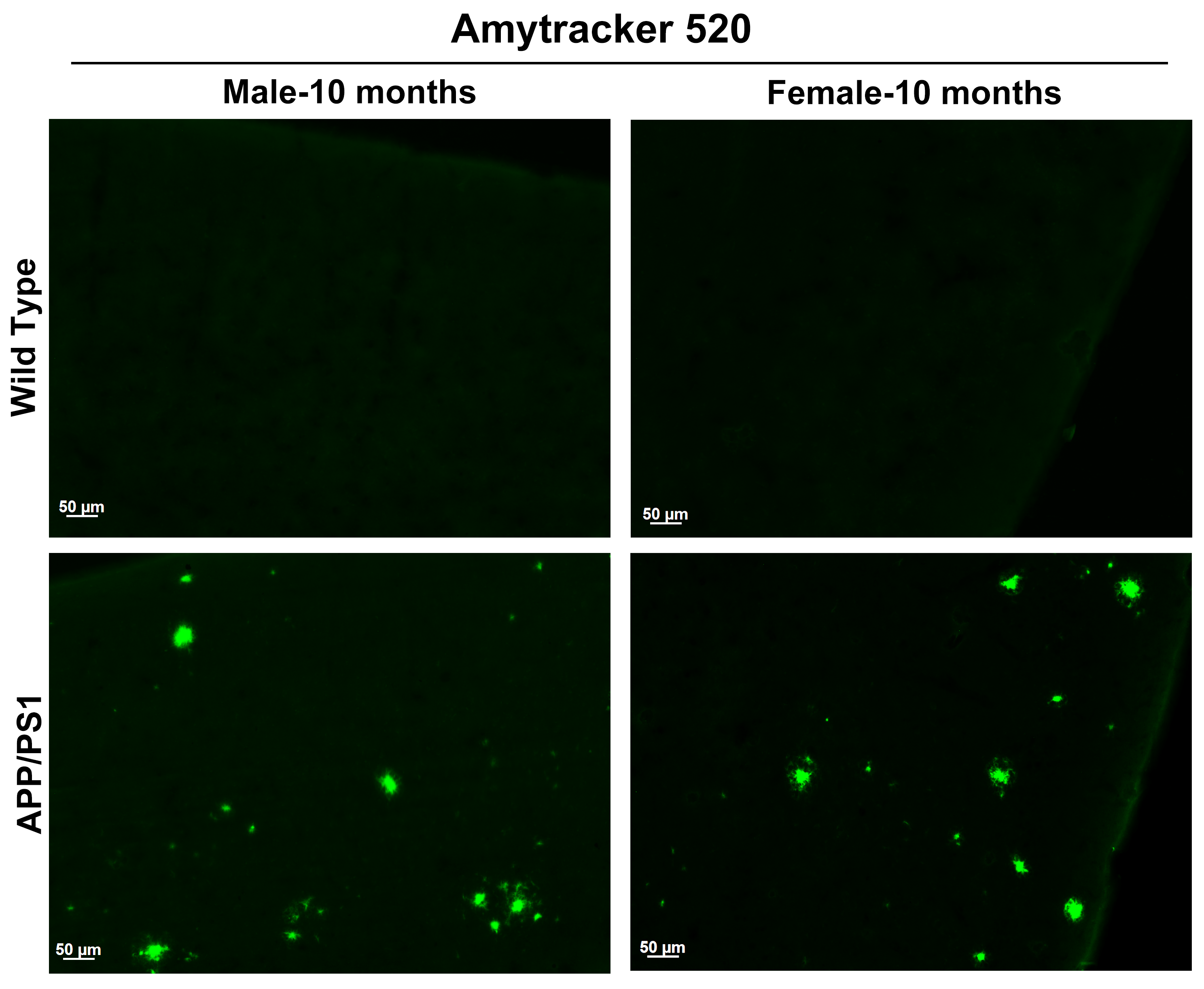

Supplement: Supplementary file 8 — Supplementary Figure. 4 [file 41398_2023_2411_MOESM8_ESM.tif]
