# Supplementary material for: Sex difference in evolution of cognitive decline: studies on mouse model and the Dominantly Inherited Alzheimer Network cohort
Source: Transl Psychiatry. 2023 Apr 12;13:123. doi: 10.1038/s41398-023-02411-8 (PMC10097702; doi:10.1038/s41398-023-02411-8)
Supplement: Supplementary file 9 — DIAN Consortia Authors List [file 41398_2023_2411_MOESM9_ESM.pdf]

DIAN Consortia Author List

LastName	First	Affiliation	Email address
Adams	Sarah	Washington University in St. Louis School of Medicine	sladams@wustl.edu
Allegri	Ricardo	Institute of Neurological Research Fleni, Buenos Aires, Argentina	rallegri@fleni.org.ar
Araki	Aki	Niigata University	araki-aki@bri.niigata-u.ac.jp
Barthelemy	Nicolas	Washington University in St. Louis School of Medicine	barthelemy.nicolas@wustl.edu
Bateman	Randall	Washington University in St. Louis School of Medicine	batemanr@wustl.edu
Bechara	Jacob	Neuroscience Research Australia	j.bechara@neura.edu.au
Benzinger	Tammie	Washington University in St. Louis School of Medicine	benzingert@wustl.edu
Berman	Sarah	University of Pittsburgh	bermans@upmc.edu
Bodge	Courtney	Brown University-Butler Hospital	Cbodge@Butler.org
Brandon	Susan	Washington University in St. Louis School of Medicine	brandons@wustl.edu
Brooks	William (Bill)	Neuroscience Research Australia	w.brooks@NeuRA.edu.au
Brosch	Jared	Indiana University	
Buck	Jill	Indiana University	jilmbuck@iu.edu
Buckles	Virginia	Washington University in St. Louis School of Medicine	bucklesv@wustl.edu
Carter	Kathleen	Emory University School of Medicine	emma.kathleen.carter@emory.edu
Cash	Lisa	Washington University in St. Louis School of Medicine	cashl@wustl.edu
Chen	Charlie	Washington University in St. Louis School of Medicine	chenc@wustl.edu
Chhatwal	Jasmeer	Brigham and Women's Hospital–Massachusetts General Hospital	Chhatwal.Jasmeer@mgh.harvard.edu
Chrem	Patricio	Institute of Neurological Research Fleni, Buenos Aires, Argentina	pchremmendez@fleni.org.ar
Chua	Jasmin	Washington University in St. Louis School of Medicine	chuajk@wustl.edu
Chui	Helena	University of Southern California	helena.chui@med.usc.edu
Cruchaga	Carlos	Washington University in St. Louis School of Medicine	cruchagac@wustl.edu
Day	Gregory S	Mayo Clinic Jacksonville	Day.gregory@mayo.edu
De La Cruz	Chrismary	Columbia University	
Denner	Darcy	Washington University in St. Louis School of Medicine	drdenner@wustl.edu

Diffenbacher	Anna	German Center for Neurodegnerative Diseases (DZNE) Munich	Anna.dieffenbacher@dzne.de
Dincer	Aylin	Washington University in St. Louis School of Medicine	aylin.dincer@wustl.edu
Donahue	Tamara	Washington University in St. Louis School of Medicine	tammie@wustl.edu
Douglas	Jane	University College London	jane.douglas@ucl.ac.uk
Duong	Duc	Emory University School of Medicine	dduong@emory.edu
Egido	Noelia	Institute of Neurological Research Fleni, Buenos Aires, Argentina	negido@fleni.org.ar
Esposito	Bianca	Icahn School of Medicine at Mount Sinai	bianca.esposito@mssm.edu
Fagan	Anne	Washington University in St. Louis School of Medicine	fanana@wustl.edu
Farlow	Marty	Indiana University	mfarlow@iupui.edu
Feldman	Becca	Washington University in St. Louis School of Medicine	rebeccaf@wustl.edu
Fitzpatrick	Colleen	Brigham and Women's Hospital-Massachusetts	cdfitzpatrick@bwh.harvard.edu
Flores	Shaney	Washington University in St. Louis School of Medicine	sflores@wustl.edu
Fox	Nick	University College London	n.fox@ucl.ac.uk
Franklin	Erin	Washington University in St. Louis School of Medicine	efranklin@wustl.edu
Friedrichsen	Nelly	Washington University in St. Louis School of Medicine	n.joseph@wustl.edu
Fujii	Hisako	Osaka City University	hfujii@med.osaka-cu.ac.jp
Gardener	Samantha	Edith Cowan University, Perth	s.gardener@ecu.edu.au
Ghetti	Bernardino	Indiana University	bghetti@iupui.edu
Goate	Alison	Icahn School of Medicine at Mount Sinai	alison.goate@mssm.edu
Goldberg	Sarah	University of Pittsburgh	goldbergs2@upmc.edu
Goldman	Jill	Columbia University	JG2673@cumc.columbia.edu
Gonzalez	Alyssa	Washington University in St. Louis School of Medicine	alyssa.gonzales@wustl.edu
Gordon	Brian	Washington University in St. Louis School of Medicine	bagordon@wustl.edu
Gräber-Sultan	Susanne	DZNE-Tübingen	susanne.graeber-sultan@dzne.de @dzne.de
Graff-Radford	Neill	Mayo Clinic Jacksonville	graffradford.neill@mayo.edu
Graham	Morgan	Mayo Clinic Jacksonville	Graham.Morgan@mayo.edu
Gray	Julia	Washington University in St. Louis School of Medicine	gray@wustl.edu

Gremminger	Emily	Washington University in St. Louis School of Medicine	egremminger@wustl.edu
Grilo	Miguel	University College London	m.grilo@ucl.ac.uk
Groves	Alex	Washington University in St. Louis School of Medicine	amgroves@wustl.edu
Haass	Christian	Ludwig-Maximilians University - Munich	Christian.Haass@mail03.med.uni-muenchen.de
Häsler	Lisa	German Center for Neurodegenerative Diseases (DZNE), Tübingen	Lisa.Haesler@dzne.de
Hassenstab	Jason	Washington University in St. Louis School of Medicine	hassenstabj@wustl.edu
Hellm	Cortaiga	Washington University in St. Louis School of Medicine	cortaiga.hellm@wustl.edu
Herries	Elizabeth	Washington University in St. Louis School of Medicine	e.herries@wustl.edu
Hoechst-Swisher	Laura	Washington University in St. Louis School of Medicine	goodl@wustl.edu
Hofmann	Anna	German Center for Neurodegenerative Diseases (DZNE), Tübingen	Anna.Hofmann@med.uni-tuebingen.de
Holtzman	David	Washington University in St. Louis School of Medicine	holtzman@wustl.edu
Hornbeck	Russ	Washington University in St. Louis School of Medicine	russ@wustl.edu
Igor	Yakushev	German Center for Neurodegnerative Diseases (DZNE) Munich	Igor.yakushev@tum.de
Ihara	Ryoko	Tokyo University	ihara-tky@umin.ac.jp
Ikeuchi	Takeshi	Niigata University	ikeuchi@bri.niigata-u.ac.jp
Ikonomovic	Snezana	University of Pittsburgh	ikonomovics@upmc.edu
Ishii	Kenji	Niigata University/Tokyo University	ishii@pet.tmig.or.jp
Jack	Clifford	Mayo Clinic Rochester	jack.clifford@mayo.edu
Jerome	Gina	Washington University in St. Louis School of Medicine	ginajerome@wustl.edu
Johnson	Erik	Emory University School of Medicine	erik.johnson@emory.edu
Jucker	Mathias	German Center for Neurodegenerative Diseases (DZNE), Tübingen	mathias.jucker@uni-tuebingen.de
Karch	Celeste	Washington University in St. Louis School of Medicine	karchc@wustl.edu
Käser	Stephan	German Center for Neurodegenerative Diseases (DZNE), Tübingen	Stephan.kaeser@uni-tuebingen.de
Kasuga	Kensaku	Niigata University	ken39@bri.niigata-u.ac.jp
Keefe	Sarah	Washington University in St. Louis School of Medicine	sarahkeefe@wustl.edu
Klunk	William (Bill)	University of Pittsburgh	klunkwe@gmail.com

Koeppe	Robert	University of Michigan	koeppe@umich.edu
Koudelis	Deb	Washington University in St. Louis School of Medicine	delanod@wustl.edu
Kuder-Buletta	Elke	German Center for Neurodegenerative Diseases (DZNE), Tübingen	elke.kuder-buletta@dzne.de
Laske	Christoph	German Center for Neurodegenerative Diseases (DZNE), Tübingen	christoph.laske@med.uni-tuebingen.de
Levey	Allan	Emory University School of Medicine	alevey@emory.edu
Levin	Johannes	German Center for Neurodegnerative Diseases (DZNE) Munich	Johannes.Levin@med.uni-muenchen.de
Li	Yan	Washington University in St. Louis School of Medicine	yanli833@wustl.edu
Lopez	Oscar	University of Pittsburgh	lopezol@upmc.edu
Marsh	Jacob	Washington University in St. Louis School of Medicine	jacobmarsh@wustl.edu
Martinez	Rita	Washington University in St. Louis School of Medicine	Ritamartinez@wustl.edu
Martins	Ralph	Edith Cowan University	r.martins@ecu.edu.au
Mason	Neal Scott	University of Pittsburgh Medical Center	masonss@upmc.edu
Masters	Colin	University of Melbourne	c.masters@unimelb.edu.au
Mawuenyega	Kwasi	Washington University in St. Louis School of Medicine	mawuenyegak@wustl.edu
McCullough	Austin	Washington University in St. Louis School of Medicine	amccullough@wustl.edu
McDade	Eric	Washington University in St. Louis School of Medicine	ericmcdade@wustl.edu
Mejia	Arlene	Columbia University	am4717@cumc.columbia.edu
Morenas-Rodriguez	Estrella	Ludwig-Maximilians University, Munich	Estrella.Morenas-Rodriguez@dzne.d
Morris	John	Washington University in St. Louis School of Medicine	jcmorris@wustl.edu
MountzMD	James	University of Pittsburgh	mountzjm@upmc.edu
Mummery	Cath	University College London	c.mummery@ucl.ac.uk
Nadkarni	Neelesh	University of Pittsburgh	nadkarnink@upmc.edu
Nagamatsu	Akemi	Tokyo University	mail:akm77-tky@umin.ac.jp
Neimeyer	Katie	Columbia University	kn2416@cumc.columbia.edu
Niimi	Yoshiki	Tokyo University	niimiy-crc@h.u-tokyo.ac.jp
Noble	James	Columbia University	jn2054@columbia.edu
Norton	Joanne	Washington University in St. Louis School of Medicine	nortonj@wustl.edu

Nuscher	Brigitte	Ludwig-Maximilians University, Munich	Brigitte.Nuscher@mail03.med.uni-muenchen.de
O'Connor	Antoinette	University College London	antoinette.o'connor@ucl.ac.uk
Obermüller	Ulricke	Hertie Institute for Clinical Brain Research	ulrike.obermueller@klinikum.uni-tuebingen.de
Patira	Riddhi	University of Pittsburgh	patirar@upmc.edu
Perrin	Richard	Washington University in St. Louis School of Medicine	rperrin@wustl.edu
Ping	Lingyan	Emory University School of Medicine	lingyan.ping@emory.edu
Preische	Oliver	German Center for Neurodegenerative Diseases (DZNE), Tübingen	Oliver.Preische@med.uni-tuebingen.de
Renton	Alan	Icahn School of Medicine at Mount Sinai	alan.renton@mssm.edu
Ringman	John	University of Southern California	john.ringman@med.usc.edu
Salloway	Stephen	Brown University-Butler Hospital	SSalloway@Butler.org
Schofield	Peter	Neuroscience Research Australia	p.schofield@neura.edu.au
Senda	Michio	Osaka City University	michio_senda@kcho.jp
Seyfried	Nick	Emory University School of Medicine	nseyfri@emory.edu
Shady	Kristine	Washington University in St. Louis School of Medicine	kesh238@g.uky.edu
Shimada	Hiroyuki	Osaka City University	h.shimada@med.osaka-cu.ac.jp
Sigurdson	Wendy	Washington University in St. Louis School of Medicine	sigurdsonw@wustl.edu
Smith	Jennifer	Washington University in St. Louis School of Medicine	smith.jennifer@wustl.edu
Smith	Lori	University of Pittsburgh	macedonials@upmc.edu
Snitz	Beth	University of Pittsburgh	snitbe@upmc.edu
Sohrabi	Hamid	Edith Cowan University	h.sohrabi@ecu.edu.au
Stephens	Sochenda	Mayo Clinic Jacksonville	Stephens.Sochenda@mayo.edu
Taddei	Kevin	Edith Cowan University	k.taddei@ecu.edu.au
Thompson	Sarah	University of Pittsburgh	thompsons24@upmc.edu
Vöglein	Jonathan	German Center for Neurodegnerative Diseases (DZNE) Munich	Jonathan.voeglein@med.uni-muenchen.de
Wang	Peter	Washington University in St. Louis School of Medicine	guoqiao@wustl.edu

Wang	Qing	Washington University in St. Louis School of Medicine	wangqing@wustl.edu
Weamer	Elise	University of Pittsburgh	weamerea@upmc.edu
Xiong	Chengjie	Washington University in St. Louis School of Medicine	chengjie@wustl.edu
Xu	Jinbin	Washington University in St. Louis School of Medicine	jinbinxu@wustl.edu
Xu	Xiong	Washington University in St. Louis School of Medicine	xxu@wustl.edu
